# Defective dimerization of FoF1‐ATP synthase secondary to glycation favors mitochondrial energy deficiency in cardiomyocytes during aging

**DOI:** 10.1111/acel.13564

**Published:** 2022-03-02

**Authors:** Diana Bou‐Teen, Celia Fernandez‐Sanz, Elisabet Miro‐Casas, Zuzana Nichtova, Elena Bonzon‐Kulichenko, Kelly Casós, Javier Inserte, Antonio Rodriguez‐Sinovas, Begoña Benito, Shey‐Shing Sheu, Jesús Vázquez, Ignacio Ferreira‐González, Marisol Ruiz‐Meana

**Affiliations:** ^1^ Cardiovascular Diseases Research Group Vall d’Hebron Institut de Recerca (VHIR) Vall d’Hebron Hospital Universitari Barcelona Spain; ^2^ Centro de Investigación Biomédica en Red de Enfermedades Cardiovasculares (CIBER‐CV) Madrid Spain; ^3^ Center for Translational Medicine Department of Medicine Thomas Jefferson University Philadelphia Pennsylvania USA; ^4^ MitoCare Center for Mitochondrial Imaging Research and Diagnostics Department of Pathology Anatomy & Cell Biol. Thomas Jefferson University Philadelphia Pennsylvania USA; ^5^ Cardiovascular Proteomics Laboratory Centro Nacional de Investigaciones Cardiovasculares Carlos III Madrid Spain

**Keywords:** aging, ATP, dicarbonyl stress, mitochondria, ROS

## Abstract

Aged cardiomyocytes develop a mismatch between energy demand and supply, the severity of which determines the onset of heart failure, and become prone to undergo cell death. The FoF1‐ATP synthase is the molecular machine that provides >90% of the ATP consumed by healthy cardiomyocytes and is proposed to form the mitochondrial permeability transition pore (mPTP), an energy‐dissipating channel involved in cell death. We investigated whether aging alters FoF1‐ATP synthase self‐assembly, a fundamental biological process involved in mitochondrial cristae morphology and energy efficiency, and the functional consequences this may have. Purified heart mitochondria and cardiomyocytes from aging mice displayed an impaired dimerization of FoF1‐ATP synthase (blue native and proximity ligation assay), associated with abnormal mitochondrial cristae tip curvature (TEM). Defective dimerization did not modify the *in vitro* hydrolase activity of FoF1‐ATP synthase but reduced the efficiency of oxidative phosphorylation in intact mitochondria (in which membrane architecture plays a fundamental role) and increased cardiomyocytes’ susceptibility to undergo energy collapse by mPTP. High throughput proteomics and fluorescence immunolabeling identified glycation of 5 subunits of FoF1‐ATP synthase as the causative mechanism of the altered dimerization. *In vitro* induction of FoF1‐ATP synthase glycation in H9c2 myoblasts recapitulated the age‐related defective FoF1‐ATP synthase assembly, reduced the relative contribution of oxidative phosphorylation to cell energy metabolism, and increased mPTP susceptibility. These results identify altered dimerization of FoF1‐ATP synthase secondary to enzyme glycation as a novel pathophysiological mechanism involved in mitochondrial cristae remodeling, energy deficiency, and increased vulnerability of cardiomyocytes to undergo mitochondrial failure during aging.

## Graphical abstract

This study identified FoF1‐ATP synthase as a prominent target of dicarbonyl stress in cardiomyocytes during aging. Non‐enzymatic glycation of several units within FoF1‐ATP synthase holocomplex is proposed to interfere with spontaneous enzyme dimerization/oligomerization, which in turn reduces cristae curvature and energy efficiency in intact mitochondria and increases the sensitivity of cardiomyocytes to undergo pathological mitochondrial permeabilization in response to stress.

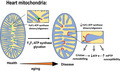

## 1 INTRODUCTION

Cardiomyocyte function relies on the regulated production of large amounts of mitochondrial ATP, to sustain their intense calcium‐dependent contractile activity throughout life. Nonetheless, the efficiency of ATP generation in cardiomyocytes declines during aging (Yaniv et al., 2013), making the heart less tolerant to exercise and stress (Peart et al., 2014) and facilitating the transition toward a failing phenotype (Neubauer, 2007). In addition, cardiac mitochondria from aged individuals are more prone to undergo energy dissipation through pathological membrane permeabilization (permeability transition pore or mPTP) (Fernandez‐Sanz et al., 2015; Hofer et al., 2009), a phenomenon causally involved in cardiomyocyte death during myocardial ischemia–reperfusion (Fernandez‐Sanz et al., 2015; Halestrap, 2010). Indeed, aged individuals have an increased cardiomyocyte death after an ischemic insult (Fernandez‐Sanz et al., 2015; Willems et al., 2005). The fact that isolated mitochondria—devoid of interaction with other cell components—from aged hearts become less efficient in generating ATP and more susceptible to undergo mPTP (Fannin et al., 1999; Fernandez‐Sanz et al., 2014) supports the existence of age‐related intrinsic mitochondrial alterations in the energy‐conserving molecular machinery.

The FoF1‐ATP synthase (mitochondrial respiratory complex V) is the universal molecule that culminates aerobic cell respiration through the generation of ATP from ADP at the inner mitochondrial membrane using the H^+^ gradient constructed by the respiratory complexes, in what is known as oxidative phosphorylation (OXPHOS). In a healthy adult heart, >90% of the ATP is produced through OXPHOS (Zuurbier et al., 2020). Self‐association of FoF1‐ATP synthase monomers into long rows of dimers drags and folds the inner membrane into cristae (Paumard et al., 2002; Strauss et al., 2008), which determine cell respiratory efficiency (Cogliati et al., 2013). When cardiomyocytes are challenged by a sustained anoxia, the FoF1‐ATP synthase paradoxically reverses into an energy‐consuming enzyme, favoring H^+^ extrusion at the expense of ATP hydrolysis (Jennings et al., 1991). Although the structure of mPTP is far from being solved, such an antagonistic role on cell energetics is in line with evidences indicating that FoF1‐ATP synthase is the molecular entity of mPTP (Alavian et al., 2014; Giorgio et al., 2013). Yet, the specific effect of aging on FoF1‐ATP synthase posttranslational modification, dimerization, and mPTP susceptibility has not been established except in a fungal model of senescence (Daum et al., 2013), in which aging induced significant disassembly of FoF1‐ATP synthase dimers, followed by profound perturbations in mitochondrial membrane structure and cell fitness.

We have recently shown increased dicarbonyl stress as a common hallmark of aging in the heart of patients and mice due to a deficient glyoxalase‐dependent detoxification system (Ruiz‐Meana et al., 2019). Dicarbonyls are highly toxic compounds that react with positively charged amino acids, promoting crosslinking, misfolding, and loss of function in certain proteins through the generation of advanced glycation end products (AGEs) (Rabbani & Thornalley, 2015). Because FoF1‐ATP synthase contains multiple positively charged amino acids in different subunits, the present study investigated FoF1‐ATP synthase as a biological target of the age‐associated dicarbonyl stress. By combining massive mass spectrometry with ultrastructural, biochemical, and functional assays, we identified an age‐dependent glycation in 5 subunits within FoF1‐ATP synthase, which impairs enzyme dimerization, reduces mitochondrial OXPHOS capacity, and has deleterious consequences on cardiomyocyte tolerance to damage.

## 2 METHODS

Cardiomyocytes, mitochondria, and myocardial extracts were obtained from young (5–6 months) and old (≥20 months) male and female C57BL/6J mice. Tissue was obtained after pentobarbital overdose administration (150 mg/Kg, i.p.). All procedures were approved by the Ethical Committee of the Vall d’Hebron Research Institute (CEEA 53/20) and comply with the EU directive 2010/63EU and Spanish transposition RD 53/2013 on protection of animals for scientific purposes.

### 2.1 Mouse myocardium

#### 2.1.1 Mass spectrometry analysis, and modified peptide and protein identification and quantification

Protein extracts were obtained by heart tissue homogenization with ceramic beads (MagNa Lyser Green Beads apparatus, Roche, Germany) in extraction buffer (50 mmol/L Tris‐HCl, 1 mmol/L EDTA, 1.5% SDS, pH 8.5). A filter‐based modification was applied for simultaneous quantitative analysis of AGE modification and of protein abundance (see Suppl. Methods).

#### 2.1.2 Transmission electron microscopy and morphometric analysis of mitochondria

Langendorff‐perfused mouse hearts were fixed, cut, and post‐fixed in 2% osmium tetroxide. Ultrathin sections were obtained with a diamond knife. For image acquisition, an FEI Tecnai 12 TEM fitted with an AMT XR‐111 10.5 Mpx CCD camera was used at 3,200–15,000× magnification (80 kV). Mitochondrial shape and mitochondrial cristae density and cristae tip curvature were quantified (Mary & Brouhard, 2019) (see Suppl. Methods).

### 2.2 Isolated mouse cardiomyocytes

Calcium tolerant rod‐shaped cardiomyocytes were isolated by Langendorff perfusion and plated on laminin‐coated glass surface (Fernandez‐Sanz et al., 2015).

#### 2.2.1 Imaging analysis of the degree of FoF1‐ATP synthase glycation

Colocalization and proximity ligation assay (PLA) were performed in fixed and permeabilized cardiomyocytes immunolabeled with anti‐ATP5a (α subunit) and anti‐methylglyoxal‐derived AGEs (MAGEs). For PLA, Duolink Insitu detection kit recommendations were followed. Z‐planes were acquired with a spectral confocal microscope (FluoView‐1000, Olympus), and positive cross‐reactivity spots were quantified in background‐subtracted 16‐bit images (Image J) (see Suppl. Methods).

#### 2.2.2 Detection of FoF1‐ATP synthase dimerization by PLA

For FoF1‐ATP synthase dimerization, PLA was performed in cardiomyocytes immunolabeled with anti‐ATP5h (d subunit), which is represented in a single copy per monomer (see Suppl. Methods).

#### 2.2.3 Susceptibility of cardiomyocytes to develop ROS‐induced mPTP

In TMRE‐loaded cardiomyocytes, ROS‐induced mPTP was determined in TMRE‐loaded cells exposed to intermittent 561 nm laser irradiation as a pH‐sensitive drop in fluorescence culminating in cell shortening (Zeiss LS980 confocal) (see Suppl. Methods).

### 2.3 Isolated heart mitochondria

Mouse heart subsarcolemmal (SSM) and interfibrillar mitochondria (IFM) were isolated by differential centrifugation (Fernandez‐Sanz et al., 2014) (see Suppl. Methods).

#### 2.3.1 Analysis of FoF1‐ATP synthase oligomerization by BN‐PAGE and in‐gel activity

Heart SSM and IFM (1.5 mg) were lysed and resolved in Native‐PAGE Bis‐Tris 3–12% gradient gel (Invitrogen, BN1001). White lead phosphate bands, indicative of ATP hydrolysis, were documented using a densitometer (GelXs Doc Quantity One, Bio‐Rad) (see Suppl. Methods).

#### 2.3.2 Western blot analysis of FoF1‐ATP synthase subunits, IF1, and OPA1

The expression of FoF1‐ATP synthase and of IF1 and OPA1 was analyzed in purified heart SSM and IFM resolved in SDS‐PAGE acrylamide gels and immunoblotted using anti‐ATP5a (subunit α), anti‐ATPb (subunit β), anti‐ATP5H (subunit d), anti‐ATP5O (subunit OSCP), anti‐IF1 (inhibitory factor 1), and anti‐OPA1 (optic atrophy 1) (see Suppl. Methods).

#### 2.3.3 ATP hydrolase activity of FoF1‐ATP synthase

*In vitro* ATPase activity sensitive to oligomycin was spectrophotometrically determined in heart SSM and IFM by two independent methods (see Suppl. Methods) (Barrientos, 2002; Novellasdemunt et al., 2013). The amount of ATP was calculated using a standard curve and normalized by mg of protein.

#### 2.3.4 Mitochondrial respiration and oligomycin‐sensitive O_2_ consumption

Mitochondrial O_2_ consumption was quantified in crude SSM and IFM using a Clark‐type electrode (Oxygraph, Hansatech) (Fernandez‐Sanz et al., 2014). Data were expressed as nmolO_2_/min*UCS (units of citrate synthase) (Fernandez‐Sanz et al., 2014) (see Suppl. Methods).

#### 2.3.5 Susceptibility of mitochondria to develop calcium‐induced mPTP

Calcium‐induced mPTP was fluorometrically determined in mitochondria subjected to consecutive calcium pulses by the quantification of their calcium retention capacity (CRC) (see Suppl. Methods).

### 2.4 Culture of H9c2 myoblasts

H9c2 cells (Sigma 88092904) were grown in Dulbecco´s Modified Eagle Medium (DMEM, ATCC, 30–2002) with 10% fetal bovine serum (FBS) and 1% penicillin/streptomycin in a saturated humidity incubator with 5% CO_2_, at 37°C. Cells were trypsinized and split at 70–80% confluence. For the experiments, cells were seeded at 20,000/cm^2^ density in 0.2% FBS medium.

#### 2.4.1 Induction of dicarbonyl stress and in vitro intracellular glycation

To simulate the dicarbonyl stress occurring in aging, H9c2 cells were plated on chambered cell culture slides and submitted to a previously standardized protocol (Ruiz‐Meana et al., 2019). Cells were treated with 5 µmol/L of glyoxalase inhibitor (SML 1306 Sigma) and 200 µmol/L methylglyoxal (MG, M0252, Sigma), and fresh supplemented medium was changed every 24 h during 3 consecutive days. A replicate of H9c2 cells grown in FBS starved culture medium was used as a control.

#### 2.4.2 Quantification of intracellular AGEs in cultured H9c2 cells

The effect of the dicarbonyl stress on intracellular AGEs was quantified by immunofluorescence and Western blot on day 3 in control and SML‐MG‐treated H9c2 cells. For immunofluorescence, fixed and permeabilized cells were immunolabeled with anti‐MAGE and Hoescht‐33342. A central Z‐plane was acquired with a spectral confocal microscope (FluoView‐1000 Olympus), and cell fluorescence was quantified in 8‐bit images (Image J). For Western blot, solubilized cells were resolved in SDS‐PAGE and immunoblotted using anti‐MAGE. Chemiluminescence was quantified with Image J (see Suppl. Methods).

#### 2.4.3 Changes in mitochondrial morphology in H9c2 cells

Time‐dependent changes in mitochondrial perimeter were quantified in H9c2 cells exposed to dicarbonyl stress (SML‐MG) or control conditions during 3 days by mitochondrial labeling with 100 nmol/L TMRE. Individual mitochondria were identified from background‐subtracted confocal images obtained at 60× (Yokogawa CSU10, Nipkow spinning disc). Mitochondrial perimeter was measured in 8‐bit images (Image J) and expressed as changes with respect to the value obtained in day 0.

#### 2.4.4 Western blot analysis of FoF1‐ATP synthase, IF1, and OPA1

The expression of FoF1‐ATP synthase and of IF1 and OPA1 was detected as in isolated heart mitochondria (see Suppl. Methods).

#### 2.4.5 Detection of FoF1‐ATP synthase glycation and dimerization in H9c2 cells by PLA

Glycation and dimerization of FoF1‐ATP synthase were determined by PLA on day 3 in control and SML‐MG‐treated H9c2 cells as described for isolated cardiomyocytes.

#### 2.4.6 Effect of glycation on FoF1‐ATP hydrolase activity in H9c2 cells

The FoF1‐ATP hydrolase activity was determined in isolated mitochondria from control and SML‐MG treated H9c2 cells on day 3 (see Suppl. Methods).

#### 2.4.7 Effect of glycation on FoF1‐ATP synthase activity in H9c2 cells

ATP production rate was monitored by real‐time ATP rate assay in a Seahorse XFp analyzer (Agilent Technologies, Seahorse Bioscience, Santa Clara, USA) in control and SML‐MG‐treated cells on day 3. Changes in the bioenergetics profile were described as the relative difference between mitoATP and glycoATP production rates compared to the total ATP rate (see Suppl. Methods).

#### 2.4.8 Spontaneous mPTP in H9c2 cells

Spontaneous time‐dependent mPTP was determined in control and SML‐MG treated H9c2 cells loaded with calcein/CoCl2 and mitotracker red (MTR) on days 0, 1, 2, and 3. Occurrence of mPTP was determined as CsA‐sensitive decay in the overlap coefficient between calcein and MTR fluorescence (Zeiss LS980) using 8‐bit images (Image J) (see Suppl. Methods).

### 2.5 Statistical analysis

Data are expressed as mean ± standard error of the mean (*SEM*). When data followed a normal distribution, a two‐tailed t‐test for independent or paired samples was applied. For data not following a normal distribution, the non‐parametric Mann–Whitney test for medians was applied. ANOVA analysis was used for comparisons between more than two groups. Differences of *p* ≤ 0.05 were considered as statistically significant. Statistical analyses were performed with SPSS v.20 software (New York, US).

## 3 RESULTS

### 3.1 Transmission electron microscopy revealed altered mitochondrial cristae morphology in the aged heart

**FIGURE 1:**
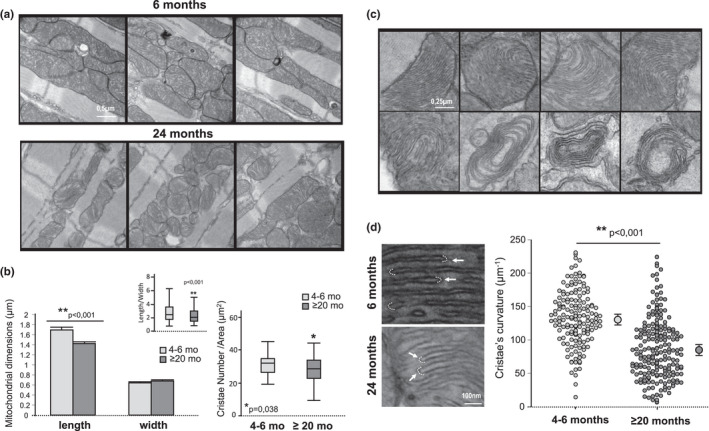
Aging alters mitochondrial cristae curvature in the heart. (a) TEM images of mouse myocardium depicting clusters of mitochondria in 6 months (upper panels) and 24 months old mice (lower panels); (b) Quantification of mitochondrial length and width (left panel) and the corresponding ratio (inset) in TEM images of mouse myocardium. Box plots (right panel) correspond to cristae number per area (µm^2^). Data correspond to *n* = 180–200 mitochondria from *n* = 3 mice per group; (c) High magnification images of mitochondria displaying a pathological pattern of varying degrees of concentrically arranged cristae, from semicircle to complete spiral (“onion‐like mitochondria”), in the myocardium of aged mice. This type of cristae morphology was not observed in young mice; (d) Cristae tip ultrastructure in a mitochondrion of a young (upper image) and an old (bottom image) mouse. Dashed lines frame representative examples of the regions of interest used to quantify cristae tip curvature. The presence of cristae with abnormal morphology and less curved tips was significantly higher in old mice. The graph shows the quantification of the curvature in *n* = 150–200 mitochondrial cristae tip per group, and the corresponding mean ± *SEM* (*n* = 3 mice per group)

A broad view of the ultrastructural images obtained with TEM allowed the identification of a large number of mitochondria with different sizes and shapes, most of them arranged along myofibrils (IFM), but also beneath the sarcolemma (SSM) in the myocardium of young and old mice (Figure 1a). Mitochondria of old mouse hearts were shorter and more rounded than those of young hearts, as quantified from mitochondrial length/width (Figure 1b). High magnification images (15,000×) revealed consistent differential pathological features specifically affecting the morphology of mitochondrial cristae in the cardiomyocytes of old mice. Mitochondrial cristae appeared less densely packed in the myocardium of aged mice, as disclosed from the significantly reduced number of crista per unit of area (µm^2^) (Figure 1c). Several IFM from the aged myocardium displayed an aberrant inner membrane folding pattern characterized by the presence of concentrically swirling cristae (“onion‐like mitochondria”) that were not observed in the mitochondria from the young myocardium (Figure 1c). Quantification of cristae tip curvature revealed a significantly lower value in the mitochondria from aged mice with respect to the young ones (Figure 1d), indicating a higher prevalence of cristae with abnormal wider angulation in their tip (i.e., looser inner membrane folding).

### 3.2 Aging impairs FoF1‐ATP synthase dimerization in cardiomyocytes

**FIGURE 2:**
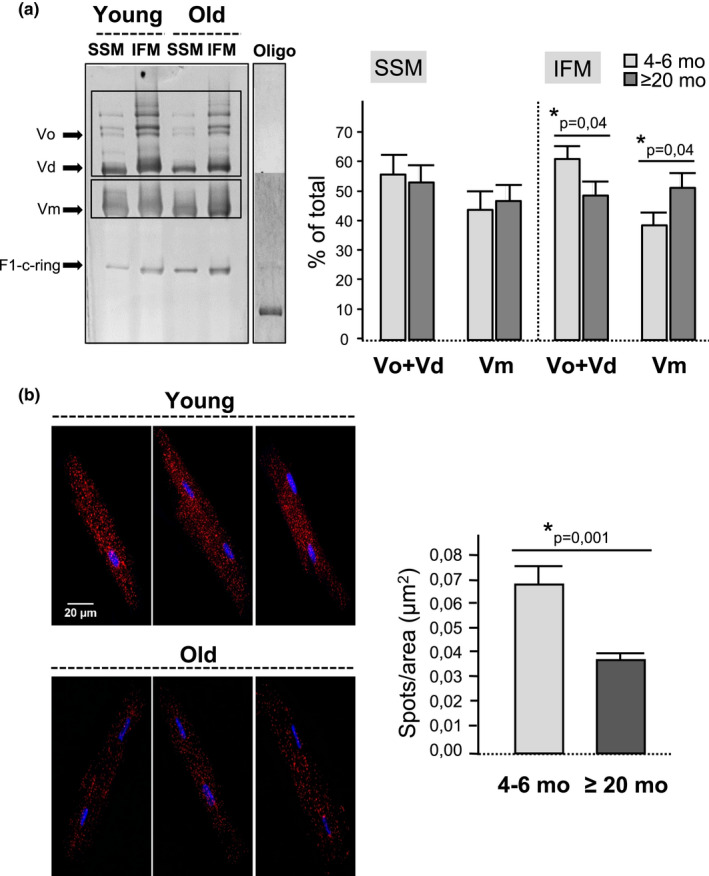
Aging impairs FoF1‐ATP synthase di‐ and oligomerization. (a) Blue native gel with precipitated lead phosphate bands corresponding to ATPase activity of the monomeric (Vm), dimeric (Vd), and oligomeric (Vo) forms of FoF1‐ATP synthase (complex V) and in the presence of FoF1‐ATP synthase inhibitor oligomycin (oligo) in digitonin‐solubilized SSM and IFM from young and old mouse hearts. Bar graph corresponds to the quantification of the optical density (OD) expressed as a percentage of ATPase activity in the monomeric and oligomeric forms with respect to the total. Data correspond to mean ± *SEM* from *n* = 6 young and *n* = 5 old mice; (b) Fluorescent confocal images of the monomer‐monomer interaction within FoF1‐ATP synthase in 6 representative cardiomyocytes (3 per group of age), detected by competitive immunolabeling against “d” subunits and PLA. Positive cross‐reactivity spots (in red) indicate enzyme dimerization, nuclei are shown in blue (Hoescht). Bar graphs correspond to the quantification of the number of amplification spots resulting from FoF1‐ATP synthase dimerization. Data correspond to mean ± *SEM* (*n* = 15–23 cardiomyocytes, *n* = 6 mice per group)

Because adequate folding of mitochondrial cristae has been shown to depend on the spontaneous assembly of FoF1‐ATP synthase monomers into long rows of dimers/oligomers that bend the mitochondrial inner membrane (Anselmi et al., 2018; Blum et al., 2019), we next investigated whether the observed alteration in mitochondrial cristae curvature can be secondary to an impaired dimerization of the FoF1‐ATP synthase in the aged heart. The degree of FoF1‐ATP synthase dimerization was assessed by (a) blue native polyacrylamide gel electrophoresis (BN‐PAGE) in purified heart mitochondria; and (b) competitive immunostaining/PLA in intact cardiomyocytes. Blue native electrophoresis of solubilized SSM and IFM followed by an in‐gel ATPase activity detected by lead phosphate precipitation disclosed a significantly lower enzyme activity in the dimeric and oligomeric forms of FoF1‐ATP synthase of the aged mice with respect to young ones that specifically affected the IFM population (Figure 2a). Nevertheless, the total mitochondrial ATPase activity (i.e., the sum of the enzyme activity in the monomeric, dimeric, and oligomeric states) remained unchanged between young and old mice, despite the age‐dependent changes in the relative contribution of the different fractions. BN‐PAGE confirmed the decrease in the abundance of the dimeric and oligomeric forms of FoF1‐ATP synthase in the IFM of old mouse hearts and the increase in the monomeric form (Figure S1). PLA using competitive immunolabeling against “d” subunit of FoF1‐ATP synthase detected a significantly lower number of positive fluorescent spots in cardiomyocytes from aged mice with respect to young ones (Figure 2b), suggesting a higher intermolecular distance between the monomeric forms of FoF1‐ATP synthase and therefore an age‐dependent decrease in FoF1‐ATP synthase dimerization. These results suggest a decreased abundance of oligomerized FoF1‐ATP synthase forms in aging.

### 3.3 FoF1‐ATP synthase abundance is preserved in the aged heart

**FIGURE 3:**
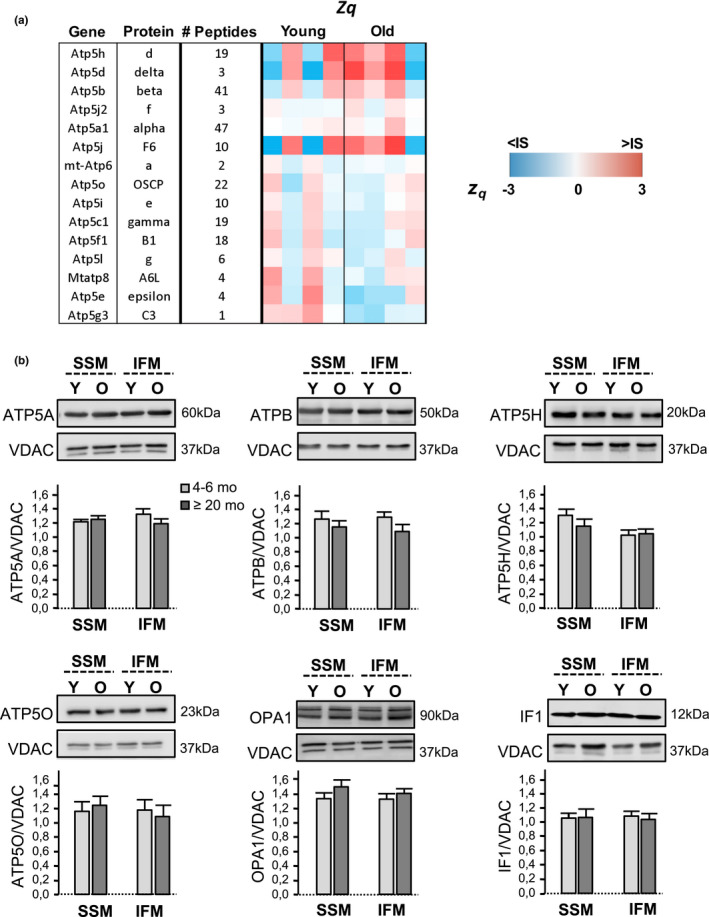
Expression of FoF1‐ATP synthase remains preserved in the aging heart. (a) Quantitation of ATP synthase subunits by MS proteomics. Data were obtained from the analysis of heart samples from young (*n* = 4) and old mice (*n* = 4). The color for each protein corresponds to its standardized protein value (zq) and is shaded according to the color scale at the bottom. IS: internal standard; (b) Abundance levels of FoF1‐ATP synthase subunits α (ATP5A), β (ATPB), d (ATP5H), and OSCP (ATP5O), and of IF1 and OPA1 proteins in purified heart SSM and IFM of young (Y) and old (O) mice, detected by Western blot; VDAC (voltage‐dependent anion channel) was used as a loading control. Bar graphs represent the ratios between the OD of each of these proteins and VDAC. Data are expressed as mean ± *SEM* (*n* = 4–6 mice per group, *p* = ns)

To investigate whether changes in the abundance levels of FoF1‐ATP synthase could underlie the age‐related reduction in the *in situ* dimerization of the enzyme, we quantified the effect of aging on the protein levels of different subunits of FoF1‐ATP synthase. Proteomics analysis of the abundance of the different subunits of the FoF1‐ATP synthase did not reveal differences in abundance between young and old mouse hearts (Figure 3a). Western blot quantification of subunits α, β, d, and OSCP of FoF1‐ATP synthase using purified SSM and IFM did not detect any age‐associated changes in the level of protein abundance (Figure 3b). Also, the abundance of the inhibitory factor 1 (IF1) and the optic atrophy 1 (OPA1), described to play a role in dimer stabilization and cristae remodeling, respectively (García et al., 2006; Varanita et al., 2015), was not modified by aging (Figure 3b). In intact cardiomyocytes, immunofluorescence labeling of α subunit of FoF1‐ATP synthase showed equal abundance pattern and fluorescence intensity in the cells from young and old mice (23 ± 2.07 and 19.59 ± 1.86 a.u.f., *p* = ns, respectively). These results consistently demonstrate that FoF1‐ATP synthase abundance remains preserved in the aged heart.

### 3.4 Several subunits of cardiac FoF1‐ATP synthase are glycated during aging

**FIGURE 4:**
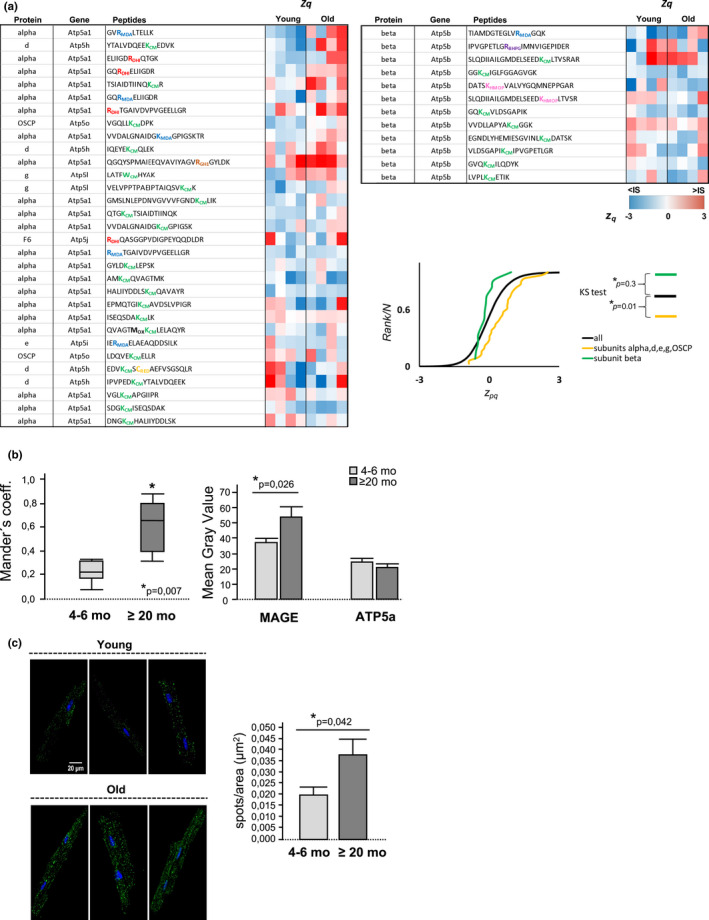
Aging increases the glycation of FoF1‐ATP synthase in cardiac mitochondria. (a) Quantitative proteomic analysis of glycated peptides from cardiac FoF1‐ATP synthase during aging. The heat‐maps show ATP synthase glycated, protein‐corrected standardized peptide zpq values, whose magnitude is shaded according to the color scale at the bottom. DHI: Dihydroxyimidazolidine; CM: Carboxymethylation; MDA: MDA adduct +54; BHPG: bis(hydroxphenylglyoxal); HMOP: 2‐ammonio‐6‐[4‐(hydroxymethyl)‐3‐oxidopyridinium‐1‐yl]‐ hexanoate; GH1: Glyoxal‐derived hydroimidazolone; RED: Carbamidomethyl (reduced) cysteine; OX: addition of one oxygen. The cumulative distributions of the zpq, (old *versus* young) values from all non‐modified peptides from all proteins (All), as well as from glycated peptides from the FoF1‐ATP synthase subunit(s) are shown. Differences were analyzed by two‐tailed Kolmogorov–Smirnov test; (b) Box plots (left panel) represent the colocalization between FoF1‐ATP synthase and MAGEs, as quantified by Mander´s coefficient of central confocal Z‐planes images in isolated immunolabeled cardiomyocytes; bar graphs (right panel) represent the gray value of the respective anti‐MAGE and anti‐ATP5A fluorescent signals in those cardiomyocyte. Data correspond to mean ± *SEM* (*n* = 16–24 cardiomyocytes, *n* = 5 mice per group); (c) Fluorescent confocal images of the interaction between FoF1‐ATP synthase and MAGE in 6 representative cardiomyocytes (3 per group of age), detected by PLA. Positive cross‐reactivity spots indicating FoF1‐ATP synthase glycation are shown in green, nuclei are shown in blue (Hoescht). Bar graphs correspond to the number of amplification spots resulting from FoF1‐ATP synthase and MAGE interaction. Data are expressed as mean ± *SEM* (*n* = 12–13 cardiomyocytes, *n* = 4 mice per group)

Non‐enzymatic glycation forms carbonyl adducts with positively charged amino acids (AGE), which may in turn impair protein structure, conformation, and activity. AGE generation is favored during aging by multiple mechanisms, including the reduction in the detoxification efficiency of methylglyoxal (MG), a highly toxic dicarbonyl intermediate (Ruiz‐Meana et al., 2019). Because FoF1‐ATP synthase contains multiple positively charged amino acids in critical enzyme subunits, we next quantified the degree of FoF1‐ATP synthase glycation in the myocardium and cardiomyocytes from young and old mice. A high‐throughput differential proteomic analysis using myocardium from young and old mice revealed a significantly higher degree of FoF1‐ATP synthase glycation in aging affecting subunits α, d, e, g, and OSCP where subunits e and g are involved in dimerization, but not in the beta subunit implicated in ATP synthesis (Figure 4a). The age‐dependent increase in FoF1‐ATP synthase glycation was corroborated in isolated cardiomyocytes by a) simultaneous immunolabeling of FoF1‐ATP synthase and methylglyoxal‐derived AGE (MAGE), which disclosed a significantly higher fluorescence colocalization in the cardiomyocytes of aged mice (Figure 4b); and b) PLA addressed to detect an intermolecular interaction <40 nm distance between MAGEs and FoF1‐ATP synthase, which revealed an increased positive cross‐reactivity in the cardiomyocytes of aged mice (Figure 4c). Overall, these results indicate that several subunits within FoF1‐ATP synthase, including those implicated in dimer formation, are targets of glycation during aging. Our data do not rule out the contribution of other mitochondrial proteins to the age‐dependent alterations in mitochondrial ultrastructure. In fact, the proteomics analysis identified 13 different peptides belonging to MICOS subunits 19, 26, 27, and 60 that can be target of dicarbonyl damage, but only 2 peptides (from Mic19 and Mic27) displayed higher levels of glycation in the aged heart with respect to the young ones (data not shown). However, none of the core MICOS subunits involved in cristae junction (i.e., Mic10 and Mic60) (Khosravi & Harner, 2020), were found to be specifically affected by the age‐dependent glycation.

### 3.5 Impact of aging on FoF1‐ATP synthase activity

**FIGURE 5:**
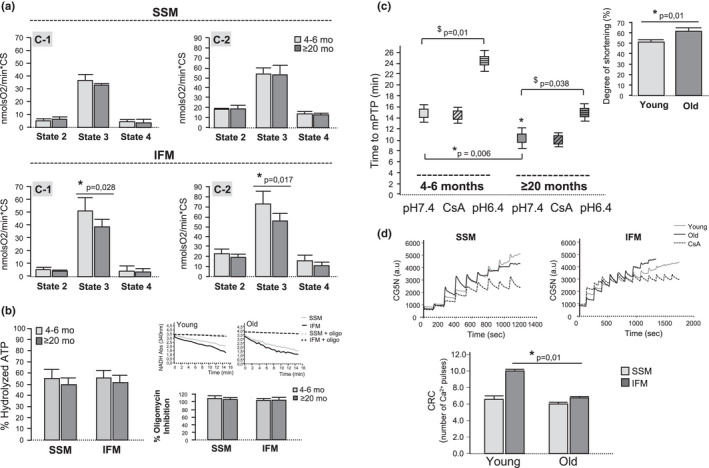
Reduced FoF1‐ATP synthase dimerization alters enzyme function in intact mitochondria and increases mPTP susceptibility. (a) Baseline oxygen consumption (state 2), ADP‐stimulated oxygen consumption (state 3), and oligomycin‐sensitive oxygen consumption (state 4) in isolated heart SSM and IFM from young and old mice, in the presence of respiratory substrates for complex 1 (C‐1) or complex 2 (C‐2), quantified by oxymetry. Data are expressed as mean ± *SEM* and correspond to nmolO_2_/min*citrate synthase (CS) (*n* = 6 mice per age group); (b) Left panel: Quantification of total ATPase activity *in vitro*, expressed as percentage of hydrolyzed ATP with respect to the total ATP, in solubilized SSM and IFM from young and old mice; Right panel (up): Kinetics of *in vitro* ATPase activity (± oligomycin) in solubilized SSM and IFM from young and old mice. Right panel (bottom): Percentage of inhibition of ATPase activity achieved after the addition of 10 µmol/L oligomycin. Data are expressed as mean ± *SEM* (*n* = 7 mice per group, p = ns); (c) Susceptibility to undergo ROS‐induced mPTP in cardiomyocytes from young and old mice, calculated as the time (in min) necessary to induce cell shortening secondary to the energetic collapse in TMRE‐loaded cells exposed to 561 nm laser illumination under control conditions (pH 7.4), and in the presence of CsA (1 µM) or acidic buffer (pH 6.4). The inset represents the degree of cell shortening secondary to mPTP in each group of age. Data are expressed as mean ± *SEM* (*n* = 18–23 cardiomyocytes, *n* = 6 mice per group); (d) Calcium retention capacity (CRC) in isolated mitochondria from old and young mouse hearts. Curves are representative examples of CG5N fluorescence throughout time upon the addition of consecutive calcium pulses (4 µmol/L) in SSM (left) and IFM (right), in the presence (or not) of 1 µmol/l CsA. Bar graphs correspond to the quantification of the average number of calcium pulses necessary to promote mPTP in both age groups and mitochondrial subpopulations. Data are expressed as mean ± *SEM* (*n* = 5 replicates per group)

The potential consequence of FoF1‐ATP synthase glycation on the enzyme activity was assessed in both intact and solubilized mitochondria (SSM and IFM) from the hearts of young and old mice. In intact mitochondria, O_2_ consumption after the addition of ADP to activate the FoF1‐ATP synthase (state 3 respiration) was significantly depressed in IFM of aged mice, regardless of the substrates used to feed the respiratory complexes (either complex 1 or 2), and despite preserved O_2_ consumption not coupled to ATP synthesis (state 2 respiration) (Figure 5a and Figure S2). This age‐dependent depression of state 3 was not observed in SSM (Figure 5a). Aging did not modify the sensitivity to the inhibitory effect of oligomycin (state 4), whose mechanism of action depends on the binding of the drug to the Fo subunit of the ATP synthase (Figure 5a). We next investigated the *in vitro* activity of FoF1‐ATP synthase using solubilized mitochondria. In this model, the contribution of the mitochondrial architecture is absent and only ATP hydrolysis can be assessed due to the lack of membrane‐associated H^+^ gradient necessary for ATP synthesis. The rate of ATP hydrolysis coupled to NADPH production in solubilized SSM and IFM from old mice remained unchanged with respect to young ones (Figure 5b). These data, together with the quantification of the total in‐gel ATP hydrolase activity using BN‐PAGE (see above), indicate that glycation of FoF1‐ATP synthase during aging does not have a direct functional impact on the enzyme; however, it reduces its efficiency to generate ATP in situ, in which the altered folding of the mitochondrial inner membrane due to less enzyme dimerization negatively impacts on OXPHOS efficiency.

### 3.6 FoF1‐ATP synthase glycation favors mitochondrial energy collapse

Because mPTP opening has been proposed to be mediated by conformational changes in FoF1‐ATP synthase (either impaired dimerization or altered c‐ring) (Alavian et al., 2014; Giorgio et al., 2013), we investigated the contribution of FoF1‐ATP synthase glycation and its decreased dimerization during aging on mPTP susceptibility using two independent approaches: ROS‐induced mPTP in intact cardiomyocytes and calcium retention capacity (CRC) in mitochondria. For ROS‐induced mPTP, TMRE‐loaded cardiomyocytes were exposed to intermittent laser irradiation, as previously validated (Ruiz‐Meana et al., 2007). The occurrence of mPTP was detected as a pH‐sensitive drop in TMRE fluorescence followed by the development of cell shortening (rigor contracture) due to energy exhaustion. In cardiomyocytes from aged mice, the time necessary to induce mPTP and cell shortening was significantly lower than in cardiomyocytes from young mice (Figure 5c); also, the degree of shortening was more pronounced in the aged cells (Figure 5c inset). The protective effect of the acidic acidic pH (6.4) in preventing mPTP was more pronounced in cardiomyocytes from young mice. CsA did not delay the occurrence of mPTP in any group of age, indicating that in this model calcium overload does not play a relevant role. For CRC, isolated SSM and IFM were exposed to consecutive calcium pulses. The occurrence of mPTP was detected as a CsA‐sensitive increase of extra‐mitochondrial CG5N fluorescence, reflecting the inability of mitochondria to reuptake calcium. The number of pulses required to induce mPTP was significantly lower in IFM from old mouse hearts with respect to IFM from young ones (Figure 5d). No age‐dependent differences in CRC were detected in SSM, although their tolerance to calcium was lower than that of IFM (Figure 5d). These results indicate that the age‐dependent changes of FoF1‐ATP synthase are associated with an increased sensitivity of cardiomyocytes to undergo mitochondrial energy collapse secondary to ROS or calcium overload. Differences in the endogenous amount of calcium in mitochondria from old mice with respect to young ones (Ruiz‐Meana et al., 2019) might also contribute to the increased sensitization toward mPTP.

### 3.7 Induction of dicarbonyl stress in H9c2 cells recapitulates age‐related changes in FoF1‐ATP synthase and favors mitochondrial failure

**FIGURE 6:**
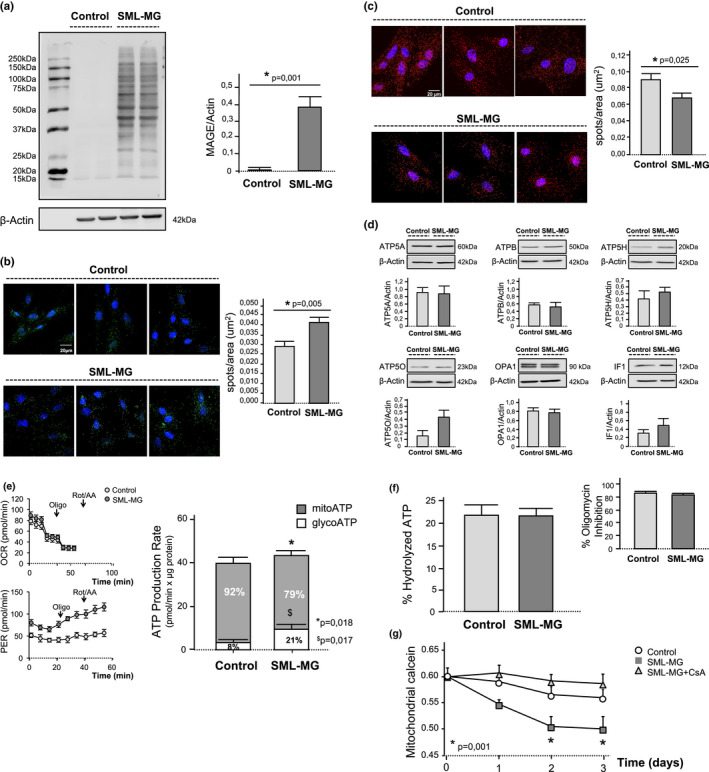
Induction of dicarbonyl stress in H9c2 cells recapitulates FoF1‐ATP synthase glycation and defective enzyme dimerization of aging, and have deleterious functional consequences on mitochondrial energetic. (a) MAGE levels in lysates from H9c2 cells on day 3 of dicarbonyl stress (5 μmol/L SML + 200 μmol/L MG) and the corresponding controls, detected by Western blot; β‐actin was used as a loading control. Bar graphs represent the ratio between the OD of the overall MAGE‐modified proteins and β‐actin in each group of cells. Data are expressed as mean ± *SEM* (*n* = 3 independent experiments); (b) Fluorescent confocal images of central Z‐planes of H9c2 cells on day 3 of dicarbonyl stress and the corresponding controls, depicting the interaction between FoF1‐ATP synthase and MAGEs as detected by PLA. Positive cross‐reactivity spots indicating FoF1‐ATP synthase glycation are shown in green, nuclei are shown in blue (Hoescht). Bar graphs correspond to the quantification of the number of amplification spots resulting from FoF1‐ATP synthase and MAGE interaction. Data correspond to mean ± *SEM* (*n* = 80–120 cells per group, 3 independent experiments); (c) Fluorescent confocal images of the monomer‐monomer interaction within FoF1‐ATP synthase in H9c2 cells on day 3 of dicarbonyl stress and the corresponding controls, detected by competitive immunolabeling against “d” subunits and PLA. Positive cross‐reactivity spots (in red) indicate enzyme dimerization, nuclei are shown in blue (Hoescht). Bar graphs correspond to the quantification of the number of amplification spots resulting from FoF1‐ATP synthase dimerization. Data correspond to mean ± *SEM* (*n* = 60–70 cell per group, 3 independent experiments); (d) Abundance levels of FoF1‐ATP synthase subunits α (ATP5A), β (ATPB), d (ATP5H) and OSCP (ATP5O), and of IF1 and OPA1 proteins in H9c2 cell extracts on day 3 of dicarbonyl stress and the corresponding controls, detected by Western blot; β‐actin was used as a loading control. Bar graphs represent the ratios of the OD of each of these proteins and β‐actin. All data are expressed as mean ± *SEM* (*n* = 3 independent experiments, p = ns); (e) ATP production rates determined by Seahorse analyzer in H9c2 cells on day 3 of dicarbonyl stress. Seahorse profiles (left) of oxygen consumption rate (OCR) and proton efflux rate (PER) in control and SML‐MG‐treated cells. The arrows indicate the points at which oligomycin (oligo) and rotenone/antimycin (Rot/AA) were injected. Bar graphs (right) represent the relative contribution of OXPHOS and glycolytic pathways, respectively, to the total rates of ATP production (pmolATP/min x µg protein) in each group; data correspond to mean ± *SEM* (*n* = 3 independent experiments (*refers to the difference in OXPHOS contribution between control and SML‐MG group, $ refers to the difference in glycolysis contribution between control and SML‐MG group); (f) ATPase activity *in vitro* in solubilized mitochondria from H9c2 cells on day 3 of dicarbonyl stress and the corresponding controls, quantified from changes in NADPH absorbance and expressed as percentage of hydrolyzed ATP with respect to the total ATP. Inset shows the percentage of inhibition of ATPase activity achieved after the addition of 1 µmol/L oligomycin. Data are expressed as mean ± *SEM* (*n* = 3 independent experiments, p = ns); (g) Spontaneous mPTP throughout 3 days in H9c2 cells exposed to dicarbonyl stress and the corresponding controls, simultaneously loaded with calcein/CoCl_2_ and mitotracker red (MTR). Occurrence of mPTP is detected as a reduction in the overlap between calcein and MTR (“mitochondrial calcein”). Data are represented as mean ± *SEM* (*n* = 3 independent experiments)

The causal relationship between intracellular dicarbonyl stress and FoF1‐ATP synthase dysfunction was investigated in H9c2 cells exposed to glyoxalase‐1 inhibition (5 µmol/L SML 1306) and methylglyoxal (200 µmol/L) for 3 days to simulate the conditions present in aged cardiomyocytes (Ruiz‐Meana et al., 2019). The chronic exposure of H9c2 cells to dicarbonyl stress significantly increased intracellular glycation at day 3, as detected by Western blot (Figure 6a) and immunofluorescence labeling of MAGEs (Figure S3). Dicarbonyl stress was associated with more mitochondrial fragmentation, quantified as time‐dependent changes in mitochondrial perimeter (Figure S4). As in aged cardiomyocytes, dicarbonyl stress resulted in a significant increase in FoF1‐ATP synthase glycation (Figure 6b). Glycation of FoF1‐ATP synthase reduced its dimerization, as detected by PLA (Figure 6c), but did not modify the expression levels of α, β, d, and OSCP subunits of FoF1‐ATP synthase in H9c2 cells, or of the IF1 and OPA1 proteins, quantified by Western blot (Figure 6d). Glycation of FoF1‐ATP synthase reduced the relative contribution of OXPHOS to ATP generation in intact cells, as disclosed by Seahorse quantification of the real‐time ATP rate production (Figure 6e), yet it did not modify the *in vitro* ATPase activity in H9c2 cells (Figure 6f). The susceptibility to undergo mPTP secondary to ROS significantly increased in H9c2 cells exposed to dicarbonyl stress (Figure S5), as in aged cardiomyocytes. Moreover, the occurrence of spontaneous mPTP throughout time, quantified as the reduction in the overlap coefficient between mitochondrial calcein and mitotracker red, was higher in H9c2 cells on days 2 and 3 in the group of dicarbonyl stress with respect to control cells (Figure 6g). Altogether, these data indicate that the induction of FoF1‐ATP synthase glycation in H9c2 cells recapitulates the reduction in the enzyme dimerization and OXPHOS capacity of the aged cardiomyocytes, and increases the sensitivity of the cells to develop mPTP in response to stress.

## 4 DISCUSSION

Exercise and stress intolerance are common characteristics of the aging heart, and their severity determines the onset of heart failure. FoF1‐ATP synthase generates >90% of the ATP (6 Kg/day) daily consumed by cardiomyocytes in a healthy heart. For an efficient ATP production, the FoF1‐ATP synthase spontaneously self‐assembles into dimers which in turn form rows of tightly ordered oligomers at the edges of cristae invaginations of the inner mitochondrial membranes (Davies et al., 2014; Dudkina et al., 2010; Hahn et al., 2016). Our study demonstrates, for the first time, that the heart of aging mice develops an impaired *in situ* dimerization of FoF1‐ATP synthase in mitochondria associated with abnormal mitochondrial cristae tip curvature, detected by TEM. High throughput proteomics and fluorescence immunolabeling identified age‐dependent glycation of several subunits of FoF1‐ATP synthase as the mechanism responsible for its impaired dimerization. This conclusion is supported by experiments in which the induction of FoF1‐ATP synthase glycation in H9c2 cells recapitulated the age‐related defective FoF1‐ATP synthase dimer assembly. Importantly, altered dimerization did not modify the in vitro activity of FoF1‐ATP synthase, but reduced its efficiency to generate H^+^ gradient‐driven ATP synthesis in intact mitochondria and increased the sensitivity of cells to undergo energy collapse through mPTP opening. Because FoF1‐ATP synthase is a fundamental energy‐converting molecular machine in the heart and has been recently proposed to be the physical entity for mPTP channel, our results identify a previously unrecognized pathophysiological mechanism involved in mitochondrial energy deficiency and cardiomyocyte death during aging.

### 4.1 Impaired dimerization of FoF1‐ATP synthase secondary to glycation reduces energy efficiency of cardiomyocytes during aging

The FoF1‐ATP synthase is the most conspicuous protein complex (~620 kDa) of the mitochondrial inner membrane, a highly invaginated bilipid structure whose architectural folding constitutes a universal mitochondrial signature evolutionarily selected to increase the efficiency of biological energy conversion (by augmenting the surface‐to‐volume ratio and serving as H^+^ traps) (Cogliati et al., 2013). In organs with elevated ATP demand, such as the heart, the mitochondrial cristae are more folded and densely packed than in less‐energy demanding organs, like the liver and kidneys (Brandt et al., 2017). Cryoelectron microscopy images reveal that FoF1‐ATP synthase, far from being randomly allocated along the membrane invaginations, aligns in arrays of V‐shaped dimers in the most tightly curved regions of the cristae (Davies et al., 2014; Dudkina et al., 2010). Studies using dynamic simulation models (Davies et al., 2012), *in vitro* reconstitution of detergent‐solubilized FoF1‐ATP synthase monomers (Blum et al., 2019) and genetically modified organisms to interfere with dimer formation (Paumard et al., 2002), unambiguously demonstrate that the spontaneous organization of FoF1‐ATP synthase monomers into dimers bends the elastic lipid bilayer (Kühlbrandt, 2015) and shapes the mitochondrial inner membrane into cristae invaginations (Bornhövd et al., 2006; Davies et al., 2012; Paumard et al., 2002; Strauss et al., 2008). In other words, mitochondrial cristae are not the structure on which FoF1‐ATP synthase dimerization occurs but the morphological consequence of dimer formation. By using native electrophoresis of SSM and IFM membranes, our study demonstrates that the relative amount of FoF1‐ATP synthase dimers/oligomers with respect to monomers is reduced in the IFM of the heart of aging mice compared to the young adults, despite no changes in the total amount of FoF1‐ATP synthase, quantified by mass spectrometry, Western blot, and immunofluorescence labeling of several independent subunits of the FoF1‐ATP synthase complex. Also, the levels of OPA1 and IF1, previously suggested to confer dimer stability (García et al., 2006; Varanita et al., 2015), were found to be preserved in the aging heart. In cardiomyocytes, the degree of monomer‐monomer interaction was addressed by competitive immunolabeling of one FoF1‐ATP synthase subunit present in a single copy per monomer followed by PLA, an approach that confirmed the existence of fewer FoF1‐ATP synthase dimers in the cells from aged mice with respect to young ones. The reduction in the relative amount of FoF1‐ATP synthase dimers/oligomers was associated with altered mitochondrial cristae morphology, detected by TEM images of myocardial tissue. In particular, several IFM of the aged myocardium exhibited a fewer number of membrane invaginations and an abnormal cristae organization characterized by a concentric arrangement (onion‐like mitochondria). The quantification of inner membrane convexity in high magnification images using an automatized b‐spline coefficient (Mary & Brouhard, 2019) disclosed a significant reduction of mitochondrial cristae tip curvature in the myocardium of aged animals compared to their young counterparts. Although the mitochondrial cristae are heterogeneous structures, the presence of swirling cristae was not observed in young mice, and studies in yeasts and cell lines have identified this morphological pattern as a hallmark of disturbed membrane folding secondary to defective FoF1‐ATP synthase dimerization (Habersetzer et al., 2013; Paumard et al., 2002). Aging was also associated with less elongated mitochondria in the myocardium, in agreement with previous studies suggesting and age‐dependent shift toward more mitochondrial fission (Amartuvshin et al., 2020).

To date, the impact of aging on FoF1‐ATP synthase dimerization had only been established in simple organismic models of senescence (Daum et al., 2013; Rampello et al., 2018; Warnsmann et al., 2021) in which altered dimer assembly was causally linked to distinct types of mitochondrial damage, including inner membrane remodeling, energy deficiency, excessive mPTP, and cell death, altogether conferring an accelerated senescence phenotype (Daum et al., 2013; Rampello et al., 2018; Warnsmann et al., 2021). In a filamentous fungus, impairing FoF1‐ATP synthase dimerization was associated with a reduced lifespan secondary to increased CyP‐D dependent mitophagy (Warnsmann et al., 2021). Nonetheless, neither the mechanism by which the dimerization of FoF1‐ATP synthase becomes defective during aging nor the consequences this may have on a complex organ like the heart have been previously addressed. Our group demonstrated that intracellular glycation (secondary to dicarbonyl stress) is a common hallmark of aging in human and murine cardiomyocytes, due to the concurrence of reduced mitochondrial antioxidant capacity and deficient glyoxalase‐dependent detoxification (Fernandez‐Sanz et al., 2014; Ruiz‐Meana et al., 2019). The ROS‐induced generation of reactive aldehydes (dicarbonyl stress) promotes advanced glycation reactions and irreversible modification of the tertiary and quaternary structure in susceptible proteins (Brownlee, 1995; Rabbani & Thornalley, 2012; Simm, 2013). However, not all proteins are targets of dicarbonyl compounds nor do all chemical reactions between dicarbonyls and amino acids necessarily carry deleterious functional consequences. FoF1‐ATP synthase has been shown to be target of transient Cys oxidation in the old murine heart (Fernandez‐Sanz et al., 2015) and lipooxidation in the aged human brain (Jové et al., 2019), pointing to the existence of a mitochondrial micro‐environment in which ROS‐induced generation of dicarbonyl compounds is thermodynamically favored. The present study confirms this prediction by immunofluorescence and mass spectrometry analysis that identified several subunits of FoF1‐ATP synthase, including those involved in the enzyme dimerization, as targets of dicarbonyl‐induced glycation during aging. These data do not allow to establish whether glycation of FoF1‐ATP synthase subunits weakens the already established contact sites within the molecule or rather impedes the assembly of FoF1‐ATP synthase monomers into dimers, therefore inverting the inner membrane curvature of the mitochondria, as previously demonstrated in yeasts (Daum et al., 2013).

Glycation of FoF1‐ATP synthase decreased mitochondrial OXPHOS capacity, as detected by a) lower ADP‐stimulated O_2_ consumption in isolated cardiac IFM, and b) compensatory shift toward glycolytic ATP production relative to oxidative ATP production in H9c2 cells. Yet the *in vitro* activity of the enzyme holocomplex (in which the contribution of mitochondrial cytoarchitecture is absent) was preserved, suggesting that monomers of FoF1‐ATP synthase are energetically self‐sufficient. Also, the sensitivity of FoF1‐ATP synthase to the inhibitory effect of oligomycin was not dependent on the glycation of the enzyme (or the relative amount of dimers), and its impact on O_2_ consumption was similar in mitochondria from aged and young animals. Altogether, these data are consistent with the concept that impaired dimerization of FoF1‐ATP synthase contributes to the reduced OXPHOS capacity of the aging heart due to changes in mitochondrial inner membrane architecture, the true bioenergetic site of cardiomyocytes. Glycation of several amino acids within FoF1‐ATP synthase is proposed to interfere with dimer assembly, as inferred from proof‐of‐concept experiments in which induction of FoF1‐ATP synthase glycation in H9c2 cells resulted in less assembled dimers and lower rate of mitochondrial ATP production. The fact that the described age‐dependent changes in FoF1‐ATP synthase distinctively affect IFM raises the question of whether the intracellular location of mitochondria and their interaction with other organelles (i.e., the sarcoplasmic reticulum) might play a critical role on their functional decline during aging.

### 4.2 Glycation of FoF1‐ATP synthase increases mPTP susceptibility

The antagonistic role of FoF1‐ATP synthase on cellular bioenergetics has long been known. The studies by Jennings et al. (Jennings et al., 1991) already established that the catalytic subunit of the FoF1‐ATP synthase reverses into an ATP hydrolase when cardiomyocytes are exposed to prolonged anoxia. This catalytic reversion precipitates ATP exhaustion in cells already jeopardized by the lack of oxygen, yet it contributes to the maintenance of mitochondrial transmembrane ionic gradients and probably represents a vestige of the endosymbiotic origin of mitochondria (Lippe et al., 2019). We have previously shown a partial failure of FoF1‐ATP synthase to revert its catalytic mode of operation in the aged cardiomyocytes that accelerates mitochondrial membrane depolarization during ischemia and impairs energy recovery upon reperfusion (Fernandez‐Sanz et al., 2015). More recently, an extreme form of energy uncoupling induced by the binding of calcium to the catalytic site of FoF1‐ATP synthase has been described, which in the presence of ROS causes a dramatic change of its structural conformation to a state whose conductance and permeability properties are similar to those of mPTP (Giorgio et al., 2017). This line of evidences postulates that FoF1‐ATP synthase is the molecular entity of the mPTP, a pathological channel whose persistent opening plays a central role in cell death during ischemia–reperfusion injury and other contexts (Baines, 2009). Either a structural alteration in the c‐ring within ATP synthase molecule (Alavian et al., 2014), a failure in FoF1‐ATP synthase dimerization (Giorgio et al., 2013) or a sequence of dimer dissociation followed by monomer rearrangement (Bonora et al., 2017) are proposed to form the energy‐dissipating channel at the inner mitochondrial membrane. We now provide evidences showing that glycation of FoF1‐ATP synthase facilitates the transition toward mPTP, an extreme energy uncoupling conformation, both in cardiomyocytes and H9c2 cells. Our data do not allow establishing the molecular entity of mPTP channel, but point to glycation‐induced changes in FoF1‐ATP synthase as the mechanism involved in the altered monomer‐monomer interface and mPTP sensitization during aging.

### 4.3 Study limitations

Because diastolic dysfunction is a prevalent and early‐onset phenotypic change in elderly patients, the lack of data on diastolic function by echocardiographic evaluation is a limitation of the present study.

## CONFLICT OF INTEREST

On behalf of all authors, the corresponding author states that there is no conflict of interest.

## AUTHOR CONTRIBUTIONS

M.R‐M performed study conceptualization, obtain funding, and prepared original draft. All authors contributed to the material preparation, data collection, analyses, interpretation of the study participated in the review and approval of the final manuscript.

## Supporting information

Supplementary Material

## Data Availability

The data, analytic methods, and study materials will be made available to other researchers on reasonable request.

## References

[acel13564-bib-0001] Alavian, K. N. , Beutner, G. , Lazrove, E. , Sacchetti, S. , Park, H.‐A. , Licznerski, P. , Li, H. , Nabili, P. , Hockensmith, K. , Graham, M. , Porter, G. A. , & Jonas, E. A. (2014). An uncoupling channel within the c‐subunit ring of the F1FO ATP synthase is the mitochondrial permeability transition pore. Proceedings of the National Academy of Sciences of the United States of America, 111(29), 10580–10585. 10.1073/pnas.1401591111 24979777 PMC4115574

[acel13564-bib-0002] Amartuvshin, O. , Lin, C.‐H. , Hsu, S.‐C. , Kao, S.‐H. , Chen, A. , Tang, W.‐C. , Chou, H.‐L. , Chang, D.‐L. , Hsu, Y.‐Y. , Hsiao, B.‐S. , Rastegari, E. , Lin, K.‐Y. , Wang, Y.‐T. , Yao, C.‐K. , Chen, G.‐C. , Chen, B.‐C. , & Hsu, H.‐J. (2020). Aging shifts mitochondrial dynamics toward fission to promote germline stem cell loss. Aging Cell, 19(8), 10.1111/acel.13191 PMC7431834 32666649

[acel13564-bib-0003] Anselmi, C. , Davies, K. M. , & Faraldo‐Gómez, J. D. (2018). Mitochondrial ATP synthase dimers spontaneously associate due to a long‐range membrane‐induced force. The Journal of General Physiology, 150(5), 763–770. 10.1085/jgp.201812033 29643173 PMC5940253

[acel13564-bib-0004] Baines, C. P. (2009). The mitochondrial permeability transition pore and ischemia‐reperfusion injury. Basic Research in Cardiology, 104(2), 181–188. 10.1007/s00395-009-0004-8 19242640 PMC2671061

[acel13564-bib-0005] Barrientos, A. (2002). In vivo and in organello assessment of OXPHOS activities. Methods, 26(4), 307–316. 10.1016/S1046-2023(02)00036-1 12054921

[acel13564-bib-0006] Blum, T. B. , Hahn, A. , Meier, T. , Davies, K. M. , & Kühlbrandt, W. (2019). Dimers of mitochondrial ATP synthase induce membrane curvature and self‐assemble into rows. Proceedings of the National Academy of Sciences of the United States of America, 116(10), 4250–4255. 10.1073/pnas.1816556116 30760595 PMC6410833

[acel13564-bib-0007] Bonora, M. , Morganti, C. , Morciano, G. , Pedriali, G. , Lebiedzinska‐Arciszewska, M. , Aquila, G. , & Pinton, P. (2017). Mitochondrial permeability transition involves dissociation of F1FO ATP synthase dimers and C‐ring conformation. EMBO Reports, 18(7), 1077–1089. 10.15252/embr.201643602 28566520 PMC5494524

[acel13564-bib-0008] Bornhövd, C. , Vogel, F. , Neupert, W. , & Reichert, A. S. (2006). Mitochondrial membrane potential is dependent on the oligomeric state of F1F0‐ATP synthase supracomplexes. The Journal of Biological Chemistry, 281(20), 13990–13998. 10.1074/jbc.M512334200 16551625

[acel13564-bib-0009] Brandt, T. , Mourier, A. , Tain, L. S. , Partridge, L. , Larsson, N.‐G. , & Kühlbrandt, W. (2017). Changes of mitochondrial ultrastructure and function during ageing in mice and Drosophila. Elife, 6, e24662. 10.7554/eLife.24662 28699890 PMC5580880

[acel13564-bib-0010] Brownlee, M. (1995). Advanced protein glycosylation in diabetes and aging. Annual Review of Medicine, 46, 223–234. 10.1146/annurev.med.46.1.223 7598459

[acel13564-bib-0011] Cogliati, S. , Frezza, C. , Soriano, M. E. , Varanita, T. , Quintana‐Cabrera, R. , Corrado, M. , Cipolat, S. , Costa, V. , Casarin, A. , Gomes, L. C. , Perales‐Clemente, E. , Salviati, L. , Fernandez‐Silva, P. , Enriquez, J. A. , & Scorrano, L. (2013). Mitochondrial cristae shape determines respiratory chain supercomplexes assembly and respiratory efficiency. Cell, 155(1), 160–171. 10.1016/j.cell.2013.08.032 24055366 PMC3790458

[acel13564-bib-0012] Daum, B. , Walter, A. , Horst, A. , Osiewacz, H. D. , & Kühlbrandt, W. (2013). Age‐dependent dissociation of ATP synthase dimers and loss of inner‐membrane cristae in mitochondria. Proceedings of the National Academy of Sciences of the United States of America, 110(38), 15301–15306. 10.1073/pnas.1305462110 24006361 PMC3780843

[acel13564-bib-0013] Davies, K. M. , Anselmi, C. , Wittig, I. , Faraldo‐Gómez, J. D. , & Kühlbrandt, W. (2012). Structure of the yeast F1Fo‐ATP synthase dimer and its role in shaping the mitochondrial cristae. Proceedings of the National Academy of Sciences of the United States of America, 109(34), 13602–13607. 10.1073/pnas.1204593109 22864911 PMC3427116

[acel13564-bib-0014] Davies, K. M. , Daum, B. , Gold, V. A. M. , Mühleip, A. W. , Brandt, T. , Blum, T. B. , Mills, D. J. , & Kühlbrandt, W. (2014). Visualization of ATP synthase dimers in mitochondria by electron cryo‐tomography. Journal of Visualized Experiments: Jove, 91, 51228. 10.3791/51228 PMC4828066 25285856

[acel13564-bib-0015] Dudkina, N. V. , Oostergetel, G. T. , Lewejohann, D. , Braun, H.‐P. , & Boekema, E. J. (2010). Row‐like organization of ATP synthase in intact mitochondria determined by cryo‐electron tomography. Biochimica Et Biophysica Acta, 1797(2), 272–277. 10.1016/j.bbabio.2009.11.004 19925775

[acel13564-bib-0016] Fannin, S. W. , Lesnefsky, E. J. , Slabe, T. J. , Hassan, M. O. , & Hoppel, C. L. (1999). Aging selectively decreases oxidative capacity in rat heart interfibrillar mitochondria. Archives of Biochemistry and Biophysics, 372(2), 399–407. 10.1006/abbi.1999.1508 10600182

[acel13564-bib-0017] Fernandez‐Sanz, C. , Ruiz‐Meana, M. , Castellano, J. , Miro‐Casas, E. , Nuñez, E. , Inserte, J. , Vázquez, J. , & Garcia‐Dorado, D. (2015). Altered FoF1 ATP synthase and susceptibility to mitochondrial permeability transition pore during ischaemia and reperfusion in aging cardiomyocytes. Thrombosis and Haemostasis, 113(3), 441–451. 10.1160/TH14-10-0901 25631625

[acel13564-bib-0018] Fernandez‐Sanz, C. , Ruiz‐Meana, M. , Miro‐Casas, E. , Nuñez, E. , Castellano, J. , Loureiro, M. , Barba, I. , Poncelas, M. , Rodriguez‐Sinovas, A. , Vázquez, J. , & Garcia‐Dorado, D. (2014). Defective sarcoplasmic reticulum‐mitochondria calcium exchange in aged mouse myocardium. Cell Death & Disease, 5, e1573. 10.1038/cddis.2014.526 25522267 PMC4454162

[acel13564-bib-0019] García, J. J. , Morales‐Ríos, E. , Cortés‐Hernandez, P. , & Rodríguez‐Zavala, J. S. (2006). The inhibitor protein (IF1) promotes dimerization of the mitochondrial F1F0‐ATP synthase. Biochemistry, 45(42), 12695–12703. 10.1021/bi060339j 17042487

[acel13564-bib-0020] Giorgio, V. , Burchell, V. , Schiavone, M. , Bassot, C. , Minervini, G. , Petronilli, V. , & Bernardi, P. (2017). Ca2+ binding to F‐ATP synthase β subunit triggers the mitochondrial permeability transition. EMBO Reports, 18(7), 1065–1076. 10.15252/embr.201643354 28507163 PMC5494526

[acel13564-bib-0021] Giorgio, V. , von Stockum, S. , Antoniel, M. , Fabbro, A. , Fogolari, F. , Forte, M. , Glick, G. D. , Petronilli, V. , Zoratti, M. , Szabo, I. , Lippe, G. , & Bernardi, P. (2013). Dimers of mitochondrial ATP synthase form the permeability transition pore. Proceedings of the National Academy of Sciences of the United States of America, 110(15), 5887–5892. 10.1073/pnas.1217823110 23530243 PMC3625323

[acel13564-bib-0022] Habersetzer, J. , Larrieu, I. , Priault, M. , Salin, B. , Rossignol, R. , Brèthes, D. , & Paumard, P. (2013). Human F1F0 ATP synthase, mitochondrial ultrastructure and OXPHOS impairment: a (super‐)complex matter? PLoS One, 8(10), e75429. 10.1371/journal.pone.0075429 24098383 PMC3788808

[acel13564-bib-0023] Hahn, A. , Parey, K. , Bublitz, M. , Mills, D. J. , Zickermann, V. , Vonck, J. , Kühlbrandt, W. , & Meier, T. (2016). Structure of a complete ATP synthase dimer reveals the molecular basis of inner mitochondrial membrane morphology. Molecular Cell, 63(3), 445–456. 10.1016/j.molcel.2016.05.037 27373333 PMC4980432

[acel13564-bib-0024] Halestrap, A. P. (2010). A pore way to die: the role of mitochondria in reperfusion injury and cardioprotection. Biochemical Society Transactions, 38(4), 841–860. 10.1042/BST0380841 20658967

[acel13564-bib-0025] Hofer, T. , Servais, S. , Seo, A. Y. , Marzetti, E. , Hiona, A. , Upadhyay, S. J. , Wohlgemuth, S. E. , & Leeuwenburgh, C. (2009). Bioenergetics and permeability transition pore opening in heart subsarcolemmal and interfibrillar mitochondria: Effects of aging and lifelong calorie restriction. Mechanisms of Ageing and Development, 130(5), 297–307. 10.1016/j.mad.2009.01.004 19428447 PMC2680750

[acel13564-bib-0026] Jennings, R. B. , Reimer, K. A. , & Steenbergen, C. (1991). Effect of inhibition of the mitochondrial ATPase on net myocardial ATP in total ischemia. Journal of Molecular and Cellular Cardiology, 23(12), 1383–1395. 10.1016/0022-2828(91)90185-o 1839801

[acel13564-bib-0027] Jové, M. , Pradas, I. , Dominguez‐Gonzalez, M. , Ferrer, I. , & Pamplona, R. (2019). Lipids and lipoxidation in human brain aging. Mitochondrial ATP‐synthase as a key lipoxidation target. Redox Biology, 23, 101082. 10.1016/j.redox.2018.101082 PMC6859548 30635167

[acel13564-bib-0028] Khosravi, S. , & Harner, M. E. (2020). The MICOS complex, a structural element of mitochondria with versatile functions. Biological Chemistry, 401(6–7), 765–778. 10.1515/hsz-2020-0103 32229686

[acel13564-bib-0029] Kühlbrandt, W. (2015). Structure and function of mitochondrial membrane protein complexes. BMC Biology, 13, 89. 10.1186/s12915-015-0201-x 26515107 PMC4625866

[acel13564-bib-0030] Lippe, G. , Coluccino, G. , Zancani, M. , Baratta, W. , & Crusiz, P. (2019). Mitochondrial F‐ATP synthase and its transition into an energy‐dissipating molecular machine. Oxidative Medicine and Cellular Longevity, 2019, 8743257. 10.1155/2019/8743257 31178976 PMC6501240

[acel13564-bib-0031] Mary, H. , & Brouhard, G. J. (2019). Kappa: Analysis of Curvature in Biological Image Data using B‐spline. BioRxiv. 10.1101/852772

[acel13564-bib-0032] Neubauer, S. (2007). The failing heart–an engine out of fuel. The New England Journal of Medicine, 356(11), 1140–1151. 10.1056/NEJMra063052 17360992

[acel13564-bib-0033] Novellasdemunt, L. , Tato, I. , Navarro‐Sabate, A. , Ruiz‐Meana, M. , Méndez‐Lucas, A. , Perales, J. C. , Garcia‐Dorado, D. , Ventura, F. , Bartrons, R. , & Rosa, J. L. (2013). Akt‐dependent activation of the heart 6‐phosphofructo‐2‐kinase/fructose‐2,6‐bisphosphatase (PFKFB2) isoenzyme by amino acids. The Journal of Biological Chemistry, 288(15), 10640–10651. 10.1074/jbc.M113.455998 23457334 PMC3624444

[acel13564-bib-0034] Paumard, P. , Vaillier, J. , Coulary, B. , Schaeffer, J. , Soubannier, V. , Mueller, D. M. , Brèthes, D. , di Rago, J.‐P. , & Velours, J. (2002). The ATP synthase is involved in generating mitochondrial cristae morphology. The EMBO Journal, 21(3), 221–230. 10.1093/emboj/21.3.221 11823415 PMC125827

[acel13564-bib-0035] Peart, J. N. , Pepe, S. , Reichelt, M. E. , Beckett, N. , See Hoe, L. , Ozberk, V. , Niesman, I. R. , Patel, H. H. , & Headrick, J. P. (2014). Dysfunctional survival‐signaling and stress‐intolerance in aged murine and human myocardium. Experimental Gerontology, 50, 72–81. 10.1016/j.exger.2013.11.015 24316036 PMC4096533

[acel13564-bib-0036] Rabbani, N. , & Thornalley, P. J. (2012). Methylglyoxal, glyoxalase 1 and the dicarbonyl proteome. Amino Acids, 42(4), 1133–1142. 10.1007/s00726-010-0783-0 20963454

[acel13564-bib-0037] Rabbani, N. , & Thornalley, P. J. (2015). Dicarbonyl stress in cell and tissue dysfunction contributing to ageing and disease. Biochemical and Biophysical Research Communications, 458(2), 221–226. 10.1016/j.bbrc.2015.01.140 25666945

[acel13564-bib-0038] Rampello, N. G. , Stenger, M. , Westermann, B. , & Osiewacz, H. D. (2018). Impact of F1Fo‐ATP‐synthase dimer assembly factors on mitochondrial function and organismic aging. Microbial Cell (Graz, Austria), 5(4), 198–207. 10.15698/mic2018.04.625 PMC5878687 29610761

[acel13564-bib-0039] Ruiz‐Meana, M. , Abellán, A. , Miró‐Casas, E. , & Garcia‐Dorado, D. (2007). Opening of mitochondrial permeability transition pore induces hypercontracture in Ca2+ overloaded cardiac myocytes. Basic Research in Cardiology, 102(6), 542–552. 10.1007/s00395-007-0675-y 17891523

[acel13564-bib-0040] Ruiz‐Meana, M. , Minguet, M. , Bou‐Teen, D. , Miro‐Casas, E. , Castans, C. , Castellano, J. , Bonzon‐Kulichenko, E. , Igual, A. , Rodriguez‐Lecoq, R. , Vázquez, J. , & Garcia‐Dorado, D. (2019). Ryanodine receptor glycation favors mitochondrial damage in the senescent heart. Circulation, 139(7), 949–964. 10.1161/CIRCULATIONAHA.118.035869 30586718

[acel13564-bib-0041] Simm, A. (2013). Protein glycation during aging and in cardiovascular disease. Journal of Proteomics, 92, 248–259. 10.1016/j.jprot.2013.05.012 23702329

[acel13564-bib-0042] Strauss, M. , Hofhaus, G. , Schröder, R. R. , & Kühlbrandt, W. (2008). Dimer ribbons of ATP synthase shape the inner mitochondrial membrane. The EMBO Journal, 27(7), 1154–1160. 10.1038/emboj.2008.35 18323778 PMC2323265

[acel13564-bib-0043] Varanita, T. , Soriano, M. E. , Romanello, V. , Zaglia, T. , Quintana‐Cabrera, R. , Semenzato, M. , Menabò, R. , Costa, V. , Civiletto, G. , Pesce, P. , Viscomi, C. , Zeviani, M. , Di Lisa, F. , Mongillo, M. , Sandri, M. , & Scorrano, L. (2015). The OPA1‐dependent mitochondrial cristae remodeling pathway controls atrophic, apoptotic, and ischemic tissue damage. Cell Metabolism, 21(6), 834–844. 10.1016/j.cmet.2015.05.007 26039448 PMC4457892

[acel13564-bib-0044] Warnsmann, V. , Marschall, L.‐M. , & Osiewacz, H. D. (2021). Impaired F1Fo‐ATP‐synthase dimerization leads to the induction of cyclophilin D‐mediated autophagy‐dependent cell death and accelerated aging. Cells, 10(4), 757. 10.3390/cells10040757 33808173 PMC8066942

[acel13564-bib-0045] Willems, L. , Zatta, A. , Holmgren, K. , Ashton, K. J. , & Headrick, J. P. (2005). Age‐related changes in ischemic tolerance in male and female mouse hearts. Journal of Molecular and Cellular Cardiology, 38(2), 245–256. 10.1016/j.yjmcc.2004.09.014 15698831

[acel13564-bib-0046] Yaniv, Y. , Juhaszova, M. , & Sollott, S. J. (2013). Age‐related changes of myocardial ATP supply and demand mechanisms. Trends in Endocrinology and Metabolism: TEM, 24(10), 495–505. 10.1016/j.tem.2013.06.001 23845538 PMC3783621

[acel13564-bib-0047] Zuurbier, C. J. , Bertrand, L. , Beauloye, C. R. , Andreadou, I. , Ruiz‐Meana, M. , Jespersen, N. R. , Kula‐Alwar, D. , Prag, H. A. , Eric Botker, H. , Dambrova, M. , Montessuit, C. , Kaambre, T. , Liepinsh, E. , Brookes, P. S. , & Krieg, T. (2020). Cardiac metabolism as a driver and therapeutic target of myocardial infarction. Journal of Cellular and Molecular Medicine, 24(11), 5937–5954. 10.1111/jcmm.15180 32384583 PMC7294140

