# Correction to “Defective Dimerization of FoF1‐ATP Synthase Secondary to Glycation Favors Mitochondrial Energy Deficiency in Cardiomyocytes During Aging”

**DOI:** 10.1111/acel.70134

**Published:** 2025-06-10

Bou‐Teen, D., Fernandez‐Sanz, C., Miro‐Casas, E., Nichtova, Z., Bonzon‐Kulichenko, E., Casós, K., Inserte, J., Rodriguez‐Sinovas, A., Benito, B., Sheu, S.‐S., Vázquez, J., Ferreira‐González, I., & Ruiz‐Meana, M. (2022). Defective dimerization of FoF1‐ATP synthase secondary to glycation favors mitochondrial energy deficiency in cardiomyocytes during aging. *Aging Cell*, 21, e13564. https://doi.org/10.1111/acel.13564

In Funding Information, the text “This work was supported by the Instituto de Salud Carlos III of the Spanish Ministry of Health (FIS‐PI19‐01196) and a grant from the Sociedad Española de Cardiología (SEC/FEC‐INV‐BAS 217003)” was insufficient.

This should have read

This work was funded by the Instituto de Salud Carlos III of the Spanish Ministry of Health (FIS‐PI19/01196) and co‐funded by the European Union (FEDER) and a grant from the Sociedad Española de Cardiología (SEC/FEC‐INV‐BAS 217003).

