# Supplementary material for: Defective dimerization of FoF1‐ATP synthase secondary to glycation favors mitochondrial energy deficiency in cardiomyocytes during aging
Source: Aging Cell. 2022 Mar 2;21(3):e13564. doi: 10.1111/acel.13564 (PMC8920436; doi:10.1111/acel.13564)
Supplement: Supplementary file 1 — Supplementary Material [file ACEL-21-e13564-s001.docx]

**SUPPLEMENTAL FIGURES**


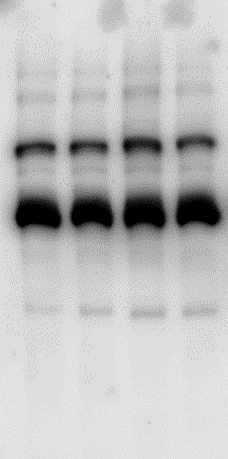


**SSM**

**IFM**

**Young**

**Old**

**Young**

**Old**

Vm

Vd

Vo

F1-c-ring

0

10

20

30

40

50

60

70

80

90

4-6 mo

≥20 mo

SSM

IFM

% of total

*p=0,044

*p=0,044

**Vo+Vd**

**Vm**

**Vo+Vd**

**Vm**

**Supplemental Figure 1: Blue native PAGE of FoF1-ATP synthase.** Representative blot labelled against subunit α of FoF1-ATP synthase showing the monomeric (Vm), dimeric (Vd) and oligomeric (Vo) forms in SSM and IFM of young and old mouse hearts. Bar graphs correspond to the quantification of the optical density (OD), expressed as a percentage of the monomeric and oligomeric forms with respect to the total. Data corresponds to mean±SEM from n=3 young and n=3 old mice.

Young

Old

**Supplemental Figure 2:** Example of an original trace of oxygen consumption (nmolO/mL) as obtained in the Oxygraph. The curve represents basal oxygen consumption after the addition of malate/glutamate (state 2), ADP-dependent oxygen consumption (state 3) and oligomycin-dependent oxygen consumption (state 4) in isolated IFM from a young and an old mouse heart.

**Control**

**SML-MG**

**Day 3**


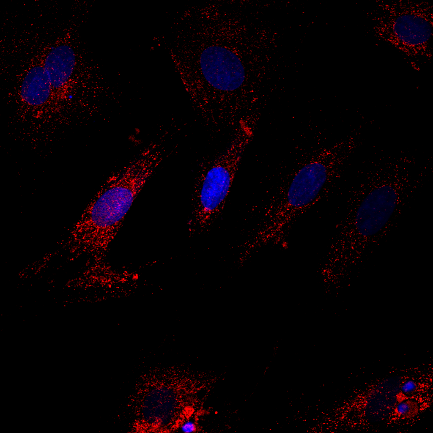

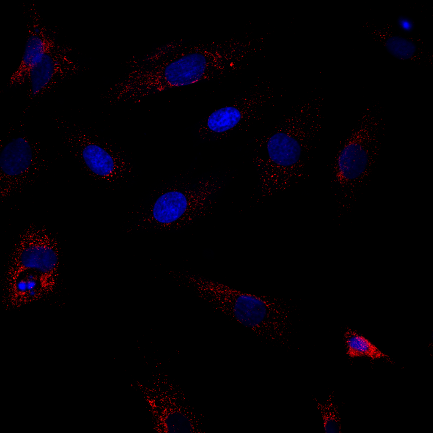

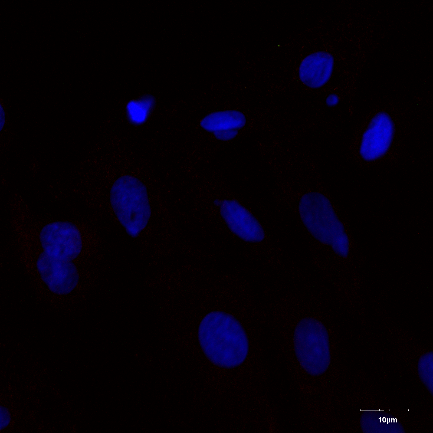

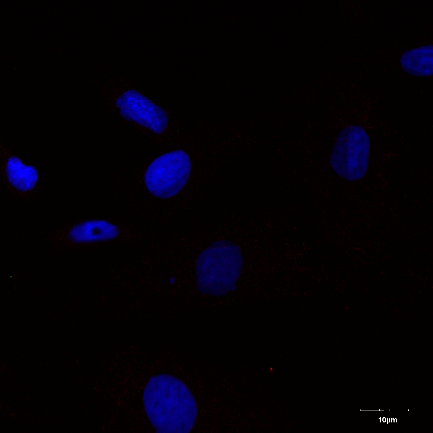


20 µm

6

4

2

0

Gray Value (a.u)

**Control**

**SML-MG**

**p<0.001

**Day 0**

**Supplemental Figure 3: Immunofluorescence of intracellular MAGEs in H9c2 cells.** Intracellular MAGES (red) in H9c2 cells on baseline (day 0) and on day 3 of dicarbonyl stress, and the corresponding controls; nuclei are shown in blue (Hoescht). Box plots represent the quantification of MAGE immunolabelling in each group (n=80-86 cells per group, 3 independent experiments).


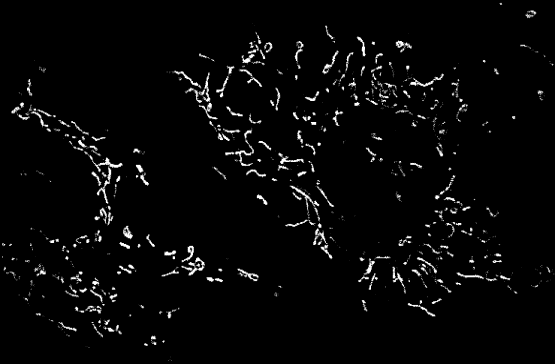


Control


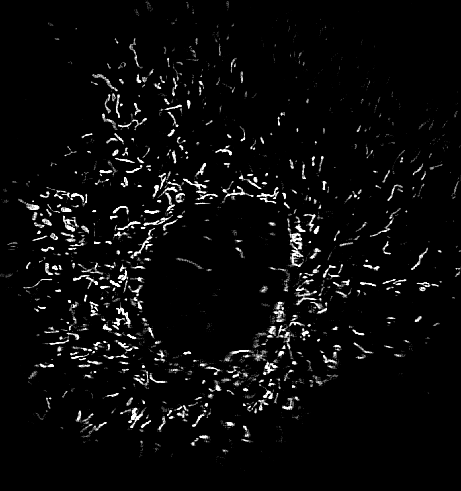

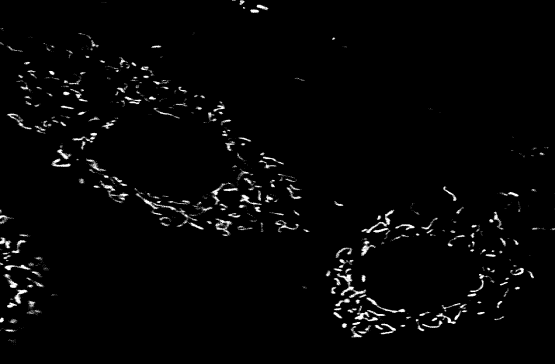


SML-MG

Control


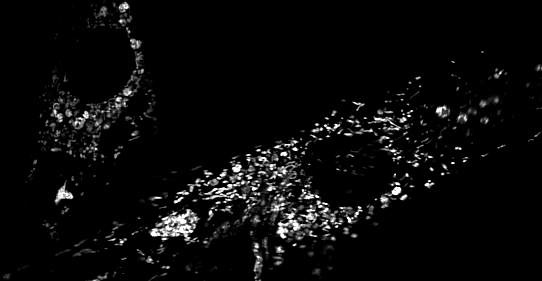


SML-MG

Day 0

Day 0

Day 3

Day 3

Control

SML-MG

0

1

2

3

0

20

40

60

80

100

* p=0,03

** p<0,001

**Time (days)**

**20um**

Control

SML-MG

Control

Day 0

Day 3

Mitochondrial perimeter

(% with respect to day 0)

**Supplemental Figure 4:** **Time-dependent changes in mitochondrial perimeter in H9c2 cells.** Representative 8-bit images of control and SML-MG treated H9c2 cells loaded with TMRE, on days 0 and 3 of dicarbonyl stress. Bar graphs correspond to the time-dependent changes in mitochondrial perimeter expressed as percentage with respect to day 0 in both groups of treatment. Data correspond to mean±SEM (n=3 independent experiments).

0

100

200

300

400

500

600

700

800

900

**CsA**

**Control**

**SML-MG**

**pH7.4**

**pH6.4**

**CsA**

**pH7.4**

**pH6.4**

*p = 0,033

^$^ p=0,013

^$^ p=0,044

Time to mPTP (sec)

**Supplemental Figure 5:** **Susceptibility to ROS-induced mPTP in H9c2 cells.** The susceptibility to undergo mPTP in control and SML-MG treated cells was estimated on day 3 as the time (in min) necessary to achieve 30% of mitochondrial membrane depolarization in TMRE-loaded cells exposed to 561nm laser illumination under control conditions (pH 7.4), and in the presence of CsA (1µmol/L) or acidic buffer (pH 6.4). Data correspond to mean±SEM (n=3 independent experiments).

**SUPPLEMENTAL METHODS**

**Mouse myocardium**

Mass spectrometry analysis, and modified peptide and protein identification and quantification

Protein extracts were obtained by mouse heart tissue homogenization with ceramic beads (MagNa Lyser Green Beads apparatus, Roche, Germany) in extraction buffer (50mmol/L Tris-HCl, 1mmol/L EDTA, 1.5% SDS, pH 8.5). Proteins were digested on-filter by the Fasilox approach using 50 mM iodoacetamide to block free (reduced) thiol groups and 40 mM DTT followed by 50 mM N-ethyl-maleimide to reduce-alkylate disulfide bonds ^21^. The resulting peptides were labeled with TMT10-plex following manufacturer instructions. The TMT10-plex experiment was composed by 4 biological replicates coming from young, 4 from old, and two channels reserved for internal standard (I.S.) samples. The I.S. was created by pooling all the samples and was used as reference to express relative quantification values. The labelled peptides were separated by high pH reversed-phase (Thermo Scientific) into 5 fractions and analyzed by nano-liquid chromatography-tandem mass spectrometry (nanoLC-MS/MS) using a FUSION mass spectrometer (Thermo Scientific). Peptide and protein identification were performed using the SEQUEST HT algorithm integrated in Proteome Discoverer 2.1 (Thermo Scientific). MS/MS scans were searched against a mouse target database (UniProtKB/Swiss-Prot, July 2016, 16958 protein sequences) including as variable modifications the following AGEs in 5 different search batches: 2-ammonio-6-[4-(hydroxymethyl)-3-oxidopyridinium-1-yl]-hexanoate (HMOP, 108.021129 Da) in Lys, hydroxyphenylglyoxal (1HPG, 132.021129 Da) in Arg, bis-hydroxphenylglyoxal (BHPG, 282.052824 Da) in Arg, dihydroxyimidazolidine in Arg (DHI, 72.021129 Da), glyoxal-derived hydroimiadazolone (G-H1, 39.994915 Da) in Arg, malondialdehyde adduct (MDA, 54.010565 Da) in Lys and Arg, and carboxymethyl (CM, 58.005479 Da) in Lys and Trp residues. Other variable modifications included Met oxidation (15.994915 Da), Cys carbamidomethylation (57.021464 Da) and methylthiolation (45.987721 Da), and TMT10-plex (229.162932 Da) on Lys and peptide N-terminus. Precursor mass tolerance was set to 800 ppm and fragment mass tolerance at 0.03 Da; precursor charge range was set to 2-4; and 3 was the maximum fragment charge. 2 miss-cleavages were allowed and only y- and b-ions were used for scoring. The same MS/MS spectra were also searched against an inverted database constructed from the same target database. False discovery rate (FDR) of peptide identifications was calculated by the refined method with an additional filter for precursor mass tolerance of 15 ppm ^22,23^. Quantitative information was extracted from MS/MS spectra of TMT-labeled peptides using Proteome Discoverer 2.1 (Thermo Scientific). Modified peptides and protein quantification was performed using the Generic Integration Algorithm ^24,25^ on the basis of the WSPP model ^26^ with some modifications ^27^. Peptide and protein abundance changes are expressed in standardized units corrected by the corresponding protein abundance (zpq) or the experiment mean (zq). Significant peptide or protein abundance changes across the different samples were detected by applying Student's t test to zpq or zq data, respectively, and differences were considered statistically significant at p < 0.05. Differences between normal distributions in terms of zpq or zq were analyzed by two-tailed Kolmogorov-Smirnov test.

Transmission electron microscopy and morphometric analysis of mitochondria

Mouse hearts were perfused in a Langendorff system with calcium Tyrode solution for 5min (in mmol/L: 135 NaCl, 5.4 KCl, 5 MgCl2, 1 CaCl2, 0.33 NaH2PO4, 10 HEPES, pH 7.3), followed by calcium free Tyrode solution and fixation buffer (2.5% glutaraldehyde in 0.15mol/L sodium cacodylate buffer, pH 7.4). Small pieces from left ventricle and papillary muscle were cut (~1 mm3) and overnight postfixed in 4°C in 2% osmium tetroxide partially reduced by 0.8% K4Fe(CN)6 in 0.15 mol/L Na-cacodylate buffer. Samples were contrasted en bloc with 1% uranylacetate in diH2O, dehydrated in graded series of acetone and embedded in Spurr’s resin. Longitudinal, ultrathin sections (65–80 nm) were cut from the resin-embedded blocks with a diamond knife (Diatome-US, USA) using a Leica UCT ultramicrotome and caught on a copper grid covered with formvar film. Images of longitudinal oriented cardiomyocytes were obtained via an FEI Tecnai 12 TEM fitted with an AMT XR-111 10.5 Mpx CCD camera at 3,200-15,000X magnification (80 kV). Cristae density was quantified as the number of cristae per area (µm^2^), and mitochondrial shape (elongated or round) was evaluated from mitochondrial length and width and from the length/width ratio measured using ImageJ in previously calibrated images. Morphometric analysis of mitochondria cristae curvature was performed using the b-spline coefficient (Kappa plugin, ImageJ) (Mary & Brouhard, 2019)

**Isolated mouse cardiomyocytes**

Calcium tolerant rod-shaped cardiomyocytes were isolated by Langendorff perfusion and plated on laminin-coated glass surface ^4^.

Imaging analysis of the degree of FoF1-ATP synthase glycation

Imaging analysis of FoF1-ATP synthase glycation was performed by colocalization and proximity ligation assay (PLA). Briefly, fixed and permeabilized cardiomyocytes from young and old mice were simultaneously immunolabeled with anti-ATP5a (ab176569 abcam, 1:100) and anti-MAGE (STA-011, Cell Biolabs, 1:50). For colocalization experiments, anti-rabbit Alexa-488 and anti-mouse Alexa-546 were used as secondary antibodies, respectively, and nuclei were counterstained with 5µg/mL Hoescht-33342. The degree of ATP5a and MAGE colocalization was determined by Mander´s correlation coefficient (Image J) in central Z-planes of 8-bit images (spectral FluorView-1000 Olympus). For PLA experiments, oligo probes-conjugated secondary antibodies (PLA probe rabbit PLUS [DUO 92002] and PLA probe mouse MINUS [DUO 92004], Sigma) were hybridized for 1h at 37°C. For ligation and amplification reactions, Duolink Insitu detection kit recommendations were followed. Z-planes were acquired with a spectral confocal microscope (FluoView-1000, Olympus). The number of positive cross-reactivity spots was quantified in background-subtracted 16-bit images (Image J).

Detection of FoF1-ATP synthase dimerization by PLA

To quantify the degree of FoF1-ATP synthase dimerization, isolated cardiomyocytes from young and old mice were immunolabeled with anti-ATP5h (d subunit, ab173006, abcam 1:100), which is represented in a single copy per monomer. Hence, the fluorescent interaction between two of these primary antibodies indicates the dimerization of two independent monomers. The detection of interaction was achieved by using equal amounts of PLUS and MINUS secondary antibodies raised against the same species. PLA oligo probes (Secondary PLA probes anti-mouse MINUS (Sigma [DUO 92004], and anti-mouse PLUS (Sigma, [DUO92001]) were hybridized for 1h at 37°C, and the ligation and amplification reactions were performed according to kit instructions. The number of positive cross-reactivity spots was quantified in background-subtracted 16-bit images (Image J).

Susceptibility of cardiomyocytes to develop mPTP

The susceptibility to undergo ROS-induced mPTP was determined in isolated cardiomyocytes from young and old mice loaded with TMRE (100nmol/L, 15min at 37ºC) and exposed to intermittent laser irradiation at 564nm (5% laser intensity, 2s interval) to induce ROS formation. CsA (1µmol/L) or pH 6.4 assay medium (in mmol/L: 140 NaCl, 20 HEPES, 1 CaCl2, 3.6 KCl, 1.2 MgSO4, 5 glucose, pH 6,4) were used in some replicates. Opening of mPTP was identified as a pH-sensitive drop in TMRE fluorescence that culminated in cardiomyocyte shortening (Zeiss LS980 confocal images) in ROIs delimiting the mitochondrial area.

**Isolated heart mitochondria**

Mouse heart subsarcolemmal (SSM) and interfibrillar mitochondria (IFM) were isolated by differential centrifugation ^8^. Briefly, heart was homogenized in cold sucrose buffer (in mmol/L: sucrose 290, MOPS 10, EGTA 1, pH 7.4) using a glass-teflon Potter-Elvehjem tissue homogenizer. The homogenate was centrifuged at 800g and the resulting supernatant at 5000g to obtain a crude fraction of SSM. The pellet obtained at 800g was resuspended in cold potassium buffer (in mmol/L: KCl 100, MOPS 50, EGTA 1, pH 7.4, 2mg of proteinase K (Sigma P2308), homogenized and subsequently centrifuged to obtain a crude fraction of IFM. For proteomic analysis and ATPase activity assays, an enriched mitochondrial fraction was obtained by an additional centrifugation (12500g) in 17% Percoll in sucrose buffer.

Analysis of mitochondrial AGEs by Western blot

To determine the effect of aging on the accumulation of mitochondrial AGEs, 35µg of heart SSM and IFM were diluted in Laemmli sample buffer 2X (S3401, Sigma), resolved in SDS-PAGE acrylamide gels and immunoblotted using anti-methylglyoxal (MG)-derived AGEs (anti-MAGE, STA-011, Cell Biolabs,1:1000). Bands were detected by chemiluminescence (Li-Cor imaging system, Odyssey) and quantified with Image J. Anti-SDHA (abcam ab14715, 1:20000) was included as an internal control.

Analysis of FoF1-ATP synthase oligomerization by BN-PAGE and in-gel activity

To analyze the effect of aging on FoF1-ATP synthase di- and oligomerization, heart SSM (0.5mg) and IFM (1.5mg) were lysed in extraction buffer (in mmol/L: 30 HEPES, 150 potassium acetate, 2 6-aminocaprionic acid, 20% glycerol, pH 7.4, 0.5mg digitonin/mg protein) and 20µg of each extract was resolved in Native-PAGE Bis-Tris 3-12% gradient gel (Invitrogen, BN1001). White lead phosphate bands, indicative of ATP hydrolysis, were observed after overnight incubation at room temperature of native gels in an ATP solution (in mmol/L: 35 Tris, 270 glycine, 14 MgSO4, 1 ATP and 0.2% lead nitrate, pH 7.8) and documented using a densitometer (GelXs Doc Quantity One, Bio-Rad). To assess oligomycin-sensitive ATPase activity, some replicates were incubated with 10µmol/L of oligomycin (495455, Millipore). Oligomers, dimers and monomers of FoF1-ATP synthase were quantified (Image J), and data were expressed as a percentage of monomers and oligomers with respect to the total.

The oligomeric state of ATP synthase solubilized with digitonin was confirmed by blue-native PAGE. Isolated SSM (0,5mg of protein) and IFM (1,5mg of protein) from hearts of young and old mice were solubilized with digitonin and extracts (20µg) were resolved on Native-PAGE Bis-Tris 3-12% gradient gel (Invitrogen, BN1001) using the same electrophoretic conditions described in BN-gels electrophoresis. After separation, the protein complexes were wet-transferred on a nitrocellulose membrane (pore size 0.45µm) using a 0.037%SDS_Bjerrum transfer buffer pH9.2 with 20% of ethanol at 20V 1h 4ºC and immunoblotted with anti-ATP5a (subunit α, ab114748 abcam, 1:3000). Bands were detected by chemiluminescence (Li-Cor imaging system, Odyssey) and quantified in Image J.

Western blot analysis of FoF1-ATP synthase subunits, IF1 and OPA1

To assess the effect of aging on the expression of FoF1-ATP synthase and of IF1 and OPA1, 30µg of heart SSM and IFM were diluted in Laemmli sample buffer 2X, resolved in SDS-PAGE acrylamide gels and immunoblotted using anti-ATP5a (subunit α, ab176569 abcam, 1:10000), anti-ATPb (subunit β, NBP2-67171 Novus Biologicals, 1:10000), anti-ATP5H (subunit d, ab173006 abcam, 1:2000), anti-ATP5O (subunit OSCP, sc-365162 SantaCruz, 1:2000), anti-IF1 (inhibitory factor 1, ab110277 abcam, 1:1000) and anti-OPA1 (optic atrophy 1, NBP1-71656 Novus Biologicals, 1:1000). Samples for ATP5O and ATP5H were previously solubilized with 1% n-dodecyl β-D-maltoside (DDM) before loading. Anti-VDAC (ab14734, abcam, 1:5000) was included as a loading control. Bands were detected by chemiluminescence and quantified with Image J.

ATP hydrolase activity of FoF1-ATP synthase

*In vitro* ATPase activity was determined in heart SSM and IFM by HK_G6PDH (H8629, Sigma) coupled ATP detecting system described previously ^20^. Briefly, crude freshly isolated SSM (1mg) and IFM (2mg) were solubilized with DDM (10mmol/L) to obtain functionally active monomers of FoF1-ATP synthase. Extracts (100µg) were incubated with ATP (in mmol/L: 2 ATP, 100 KCl, 50 MOPS, pH 7.4) for 15min at room temperature to allow ATP hydrolysis and the reaction was stopped with 10µmol/L of oligomycin. For each sample, a replicate with oligomycin (10µmol/L) present from the beginning of the reaction was added to obtain 100% of ATP (total inhibition of ATP hydrolysis). Non-hydrolysed ATP was determined from 50µL of each sample in HK_G6PDH solution (in mmol/L: 2.5 glucose, 2 NAPD, and 2U of HK_G6PDH) and the increase in absorbance at 340nm of NADPH was monitored (iD3 multimode plate reader, Molecular Devices). The amount of ATP was calculated using a standard curve (range: 0-4 mmol/L ATP) and normalized by mg of protein. For the kinetics of ATP hydrolysis, after 15min incubation in DDM, 20-40µg of protein was diluted in 200µL of assay buffer containing in mmol/L: 50 MOPS (pH 7.4), 100 KCl, 1 EGTA, 1 MgCl_2_, phospho-enol pyruvate (PEP, 11444257 Fisher Scientific), 1.3 NADH (43420, Sigma) and 30U/mL pyruvate kinase_lactate DH (P0294-5ML, Sigma). The reaction started after addition of MOPS-buffered ATP (final concentration 2mmol/L). A replicate for each condition was incubated with oligomycin 1µmol/L in order to assess whole correctly assembled FoF1-ATP synthase sensitive to oligomycin.

Mitochondrial respiration and oligomycin-sensitive O2 consumption

Mitochondrial O2 consumption was quantified in crude SSM and IFM from hearts of young and old mice using a Clark-type electrode (Oxygraph, Hansatech) as described ^8^. Non-ADP dependent O2 consumption (state 2) was monitored by using substrates for complex 1 (2mmol/L malate and 5mmol/L glutamate) or complex 2 (5mmol/L succinate with 0.5μmmol/L rotenone). ADP-stimulated O2 consumption (state 3) was achieved after addition of 250μmol/L ADP. Before ADP exhaustion, 1µmol/L of oligomycin was added to inhibit FoF1-ATP synthase (state 4). Data were expressed as nmolO2/min*UCS (units of citrate synthase) where USC was determined as nmols TNB/min x mg of mitochondrial protein ^8^.

**Culture of H9c2 myoblasts**

H9c2 cells (Sigma 88092904) were grown in high glucose Dulbecco´s Modified Eagle Medium (DMEM, ATCC, 30-2002) supplemented with 10% fetal bovine serum (FBS) and 1% penicillin/streptomycin in a saturated humidity incubator with 5% CO2, at 37ºC and used between passages 8-15. Cells were trypsinized and split at 70-80% confluence. For the experiments, cells were seeded at 20000/cm2 density in 0.2% FBS medium.

Induction of dicarbonyl stress and in vitro intracellular glycation

To simulate the dicarbonyl stress occurring in aging, H9c2 cells were plated on chambered cell culture slides and submitted to a previously standardized protocol ^18^. Cells were treated with 5µmol/L of glyoxalase inhibitor (SML-1306 Sigma) and 200µmol/L methylglyoxal (MG, M0252, Sigma), and fresh supplemented medium was changed every 24h during 3 consecutive days. A replicate of H9c2 cells grown in FBS starved culture medium was used as a control.

Quantification of intracellular AGEs in cultured H9c2 cells

To determine the effect of the dicarbonyl stress on the accumulation of AGEs, immunofluorescence and Western blot analysis were performed on day 3 in control and SML-MG treated H9c2 cells. For immunofluorescence, cells were fixed (4% formaldehyde), permeabilized (0.1% Triton X-100) and immunolabeled with anti-MAGE antibody (HM5014, Hycult biotec, 1:50). Nuclei were counterstained with 5µg/mL Hoescht-33342. A central Z-plane image was acquired with a spectral confocal microscope (FluoView-1000 Olympus) and total cell fluorescence was quantified as mean gray value in 8-bit images (Image J). For Western blot, control and SML-MG treated cells on day 3 were solubilized in lysis buffer (in mmol/L: 50 Tris HCl, 10 EDTA, 150 NaCl, 1% Triton X-100, DDT, 10 sodium fluoride, 2 sodium orthovanadate, 1% protease inhibitor cocktail [P8340, Sigma]). Extracts (50µg) were resolved in SDS-PAGE and immunoblotted using anti-MAGE (STA-011Cell Biolabs, 1:1000). Anti-β-actin (A5441Sigma, 1:5000) was used as a loading control. Bands were detected by chemiluminescence and quantified with Image J.

Western blot analysis of FoF1-ATP synthase, IF1 and OPA1

To assess changes in abundance in FoF1-ATP synthase, IF1 and OPA1 in response to dicarbonyl stress, cell lysates (50µg) from control and SML-MG treated cells on day 3 of treatment were resolved in SDS-PAGE electrophoresis and immunoblotted using the same experimental conditions described in isolated heart mitochondria. Anti β-actin (Sigma A5441, 1:5000) was used as a loading control. Bands were detected by chemiluminescence and quantified with Image J.

Detection of FoF1-ATP synthase glycation and dimerization in H9c2 cells by PLA

The effect of dicarbonyl stress on the degree of glycation and dimerization of FoF1-ATP synthase in H9c2 cells was determined by PLA on day 3 of glycative treatment in cells plated on chambered culture slides using the same protocol as described for isolated mouse cardiomyocytes.

Effect of glycation on FoF1-ATP hydrolase activity in H9c2 cells

To determine the effect of glycation on the FoF1-ATP hydrolase activity, mitochondria were isolated from control and SML-MG treated H9c2 cells (five p100 plates) on day 3 of glycative treatment. Trypsinzed cells were homogenized (glass-teflon Potter-Elvehjem homogenizer) in cold sucrose isolation buffer with 5U DNase (04536282001, ROCHE) and 10U proteinase K and differentially centrifuged. The FoF1-ATP hydrolase activity was determined from 20µg of DDM (1%) solubilized mitochondria using the same protocol described for heart mitochondria.

Effect of glycation on FoF1-ATP synthase activity in H9c2 cells

To assess the effect of glycation of FoF1-ATP synthase on the efficiency of mitochondrial ATP generation (i.e., the relative contribution of OXPHOS with respect to glycolytic pathways), ATP production rate was monitored by real-time ATP rate assay (103591-100, Agilent) in a Seahorse XFp analyzer (Agilent Technologies, Seahorse Bioscience, Santa Clara, USA). Control and SML-MG treated cells were trypsinized on day 3 of glycative treatment and seeded at 15000 cells/well in culture media (0.2%FBS ± 5µmol/L SML-200µmol/L MG) in Seahorse XF HS Mini 8-well plates (103022-100) precoated with 0.1% gelatin, at 37ºC. After 24h, culture medium was replaced by DMEM medium pH 7.4 (103575-100, Agilent) supplemented with 25mmol/L glucose, 1mmol/L pyruvate and 2mmol/L L-glutamine and incubated at 37ºC (1h, non-CO2 incubator). Real-Time ATP rate assay was performed using 1.5 µmol/L oligomycin, 0.5 µmol/L rotenone and 0.5 µmol/L antimycin A. At the end of the experiment, cells were lysed (0.1% Triton X-100) and protein was determined by Bradford. The ATP production rate was calculated from the rate of O2 consumption coupled to ATP production (OCRATP) during OXPHOS (mitoATP) and from lactate production during glycolysis (ECAR data), which was converted to glycolytic ATP production rate (glycoATP). MitoATP and GlycoATP rates (pmols/min) were normalized by µg of protein. Changes in the bioenergetics profile as a consequence of in vitro dicarbonyl stress were described as the relative difference between mitoATP and glycoATP production rates compared to the total ATP rate.

Susceptibility of H9c2 cells to develop ROS-induced mPTP

The effect of in vitro FoF1-ATP synthase glycation on ROS-induced mPTP susceptibility was determined in TMRE-loaded H9c2 cells as in isolated cardiomyocytes. Occurrence of mPTP was quantified on day 3 of the glycative treatment as a pH-sensitive 30% drop of TMRE fluorescence.

Spontaneous mPTP and cell death in H9c2 cells

The impact of dicarbonyl stress on spontaneous time-dependent mPTP was determined in control and SML-MG treated H9c2 cells simultaneously loaded with calcein (1µmol/L, 15min, 37ºC) and mitotracker red (MTR, 200nmol/L, 30min, 37ºC) on days 0, 1, 2 and 3. Cytosolic calcein fluorescence was quenched with 1mmol/L of CoCl2 (15min, 37ºC). In some replicates, 0.2µmol/L CsA was present during dicarbonyl stress induction. Occurrence of mPTP was determined on days 0, 1, 2 and 3 as CsA-sensitive decay in the overlap coefficient between calcein and MTR fluorescence (Zeiss LS980) using 8-bit images (Image J). The potential consequence of mPTP on cell viability were measured by thiazolyl blue tetrazolium bromide assay (MTT, Sigma, M2128) on days 0, 1, 2 and 3 in control and SML-MG treated H9c2 cells. At each time point, 0.5mg/mL MTT was added to the cells and incubated for 3h (37°C, 5% CO2). The formazan crystals were solubilized with DMSO (30min, 37ºC) and cell death was expressed as changes in the absorbance at 620nm (multiplate reader Multiscan FC, ThermoScientific) on days 1, 2 and 3 with respect to day 0.
